# Essential Amino Acid Intake Is Required for Sustaining Serum Insulin-like Growth Factor-I Levels but Is Not Necessarily Needed for Body Growth

**DOI:** 10.3390/cells11091523

**Published:** 2022-05-02

**Authors:** Hiroki Nishi, Kaito Uchida, Maki Saito, Daisuke Yamanaka, Haruka Nagata, Hinako Tomoshige, Ichiro Miyata, Koichi Ito, Yuka Toyoshima, Shin-Ichiro Takahashi, Fumihiko Hakuno, Asako Takenaka

**Affiliations:** 1 Department of Animal Resource Sciences, Graduate School of Agricultural and Life Sciences, The University of Tokyo, Tokyo 113-8657, Japan; onepiece1990914@gmail.com (H.N.); harukan.74@gmail.com (H.N.); hinako4t@gmail.com (H.T.); 2 Department of Agricultural Chemistry, School of Agriculture, Meiji University, Kawasaki 214-8571, Japan; baronkite0923@gmail.com; 3 Department of Pediatrics, Jikei University School of Medicine, Tokyo 105-8461, Japan; msaito@jikei.ac.jp (M.S.); i-miyata@jikei.ac.jp (I.M.); 4 Department of Food and Physiological Models, Graduate School of Agriculture and Life Sciences, The University of Tokyo, Kasama 319-0206, Japan; adyama@mail.ecc.u-tokyo.ac.jp (D.Y.); akoito@mail.ecc.u-tokyo.ac.jp (K.I.); 5 School of Agriculture, Utsunomiya University, Utsunomiya 321-8505, Japan; yukat@cc.utsunomiya-u.ac.jp

**Keywords:** essential amino acid (EAA), growth, insulin-like growth factor I (IGF-I), growth hormone (GH)

## Abstract

Essential amino acids (EAAs) are those that cannot be synthesized enough to meet organismal demand; therefore, it is believed that they must be taken from the diet for optimal growth. The growth hormone (GH)/insulin-like growth factor-I (IGF-I) system is also considered significant for growth regulation in mammals. This study aimed to evaluate the relative contributions of protein nutrition and the GH/IGF-I system to body growth regulation. Experiments using rodents and hepatocyte-derived cell lines subjected to EAA deficiency showed that a reduction in the serum EAA concentration hinders *Igf1* transcription in the liver in a cell-autonomous manner, thereby decreasing serum IGF-I levels. Remarkably, when the serum IGF-I level of mice on a low-protein diet was restored by the recombinant IGF-I infusion, the body growth was mostly rescued, although the mice were still deficient in EAA intake. Meanwhile, the GH signal activation and subsequent *Igf1* transcription were also dramatically diminished by EAA deprivation in the cell culture model. Altogether, we demonstrate that EAAs are not strictly necessary for animal growth as building blocks but are required as IGF-I-tropic cues. The results will bring a paradigm shift regarding the definition of “essential” amino acids.

## 1. Introduction

Essential amino acid (EAA) is an amino acid that cannot be synthesized in an organism enough to meet its demand. Therefore, it is believed that EAAs have to be taken from the diet to maintain body homeostasis. EAAs were first identified in the 1930s by William C. Rose and his colleagues as amino acids without which animals cannot grow adequately, lose weight, and eventually die [1]. He also established the minimum daily requirements of each amino acid for optimal growth using rats and humans [1]. Even today, we still appreciate these observations and consider them to be the standard knowledge of protein nutrition.

When growing animals are reared on a low-protein diet or EAA-deficient diet, the whole-body protein turnover will be lowered, and body protein deposition, especially in the skeletal muscle, will significantly decrease, leading to body weight loss and growth retardation [2,3,4]. Given that approximately 40–50% of the dry weight of the mammalian body consists of proteins [5], growth retardation from protein malnutrition is believed to be due to the lack of building blocks. On the other hand, it is well established that GH/IGF-I system is closely involved in growth regulation [6]. Growth hormone, which is produced and secreted by the anterior pituitary, goes through the circulation to the liver, where it stimulates the GH receptor/Janus kinase (JAK) 2/signal transducer and activator of transcription (STAT) 5 pathway to promote IGF-I production [7]. Both GH and IGF-I systemic knockout mice showed severe defects in development and growth, and the disruption of the osteoblast IGF-I signaling suppressed bone growth [8,9]. In addition, rodents fed a low-protein diet exhibited decreased GH and IGF-I levels in the serum [10,11] and developed GH resistance [12], suggesting that protein nutrition has a considerable impact on GH/IGF-I axis. These findings indicate that dietary protein, GH/IGF-I axis, and body growth are tightly interrelated.

The current study aimed to evaluate the relative contributions of protein nutrition and the GH/IGF-I system to body growth regulation. Here, we show that recombinant IGF-I infusion to mice fed a low-protein diet significantly reversed growth retardation, although their protein requirement was still not satisfied. These surprising results will bring a paradigm shift regarding the definition of “essential” amino acids.

## 2. Materials and Methods

### 2.1. Materials

For the experimental animal diets, the vitamin mixture, mineral mixture, cellulose powder, and corn starch were purchased from the Oriental Yeast Co. (Tokyo, Japan). At the same time, the soybean oil was acquired from the Nacalai Tesque (Tokyo, Japan), and the corn oil and sucrose were acquired from the Fujifilm Wako Pure Chemical Corporation (Osaka, Japan). Recombinant human IGF-I was obtained from Astellas Pharma Inc. (Tokyo, Japan). Nutrient Mixture F-12 Ham (Ham’s F12, #F6636, Sigma, St. Louis, MO, USA), William’s E medium (#W4125), 10× Earle’s buffered salt solution (EBSS), 100× MEM vitamin solution, and fetal bovine serum (FBS) were purchased from the Sigma Aldrich (St. Louis, MO, USA). Dulbecco’s modified Eagle’s medium (DMEM) was purchased from the Nissui Pharmaceutical Co. (Tokyo, Japan). Human recombinant growth hormone (Growject^®^) was obtained from the JCR Pharmaceuticals (Hyogo, Japan). Other reagent-grade chemicals used in this study were commercially available.

### 2.2. Animals

Wistar rats were purchased from the Charles River Japan (Kanagawa, Japan). The rats were caged individually and maintained at 24 ± 1 °C with 50–60% humidity and under a 12-h light/dark cycle (8:00–20:00/20:00–8:00). They were allowed free access to food and water.

Prior to the experiments, five-week-old male rats were fed normal chow for 3 days and a control (CN) diet containing 15% (*w*/*w*) amino acids (Table S1) for the next 4 days as a prefeeding period. The animals were divided into experimental groups. After that, each group was given either the CN diet or each experimental diet for 7 days. In the low-amino acid diet (5AA) and a single amino acid-deficient diet, the concentration of all amino acids or only the indicated amino acid was 1/3 of that of the CN diet. All the diets were self-made. Throughout the experimental period, all rats’ body weight and food intake were measured at 10:00 a.m. every day. On the last day, the rats were anesthetized with isoflurane (DS Pharma Animal Health, Tokyo, Japan) and then decapitated. Blood samples were collected from the carotid arteries, and the livers were collected. Serum was prepared immediately after blood collection, and liver samples were immediately frozen in liquid nitrogen. All samples were stored at −80 °C for future use.

Seven-week-old C57BL/6 male mice were purchased from the Sankyo Labo Service Corporation, Inc. (Tokyo, Japan), kept at 22–24 °C under a 12-h light/dark cycle (6:00–18:00/18:00–6:00), and allowed free access to tap water throughout the experiment. Prior to the experiment, mice were fed ad libitum a normal chow and then fed a control diet (CON) (Table S2) for 3 days before implanting an ALZET osmotic pump model 1002 (0.25 µL/h) (Durect Corp, Cupertino, CA, USA). From the day of pump implantation, either 40 µg/day of rhIGF-I or vehicle (0.1 M acetic acid) was continuously administered, and either a control diet (CON) containing 15% protein or a low-protein diet (LP) containing 3% protein was given for 10 days. After fasting for 12–15 h on the last day of the experiment, the mice were dissected under isoflurane anesthesia, circulating blood was collected from the heart, and organs were collected. Heparinized plasma and organs were stored at −80 °C until use.

All animal care and experiments conformed to the Guidelines for Animal Experiments of the University of Tokyo and Meiji University. They were approved by the Animal Research Committee of The University of Tokyo and the Meiji University Institutional Animal Care and Use Committee.

### 2.3. Blood Parameters

The IGF-I concentrations in the serum and plasma were measured using a mouse/rat IGF-I Quantakine ELISA kit and IGF-I Human ELISA Kit (DG 100) (R&D Systems, Minneapolis, MN, USA).

According to previous procedures, plasma 3-methyl-L-histidine concentration was measured with some modifications [13]. To 20 µL of plasma, 20 µL of internal standard (5 nmol/mL 1-Methyl-L-Histidine) and 40 µL of 20% trichloroacetic acid were added. After centrifugation (4 °C, 12,000× *g*) for 10 min, 120 µL of diethyl ether was added to the supernatant, and the mixture was centrifuged (4 °C, 12,000× *g*) for 1 min. The recovered water phase was mixed with 200 µL of milli Q water and half-volume of OPA reagent (25 mg o-phthalaldehyde, 0.5 mL methanol, 1.5 mL 0.4 M borate buffer, pH 12.0) and then subjected to HPLC analysis. The analytical column used was a PEGASIL ODS SP100 (Senshu Kagaku, Tokyo, Japan). The column temperature was 40 °C, with a mobile phase of acetonitrile/50 mM sodium acetate buffer (pH 5.0; 10:90, *v*/*v*), and detection was performed using a fluorescence detector (Ex 340 nm, Em 455 nm).

For the serum amino acid analysis, 50 µL of the serum samples were mixed on ice with 120 μL methanol containing internal control substances: 25 μM 2-morpholinoethanesulfonic acid and 100 μM methionine sulfone. After centrifugation (16,000× *g* for 10 min at 4 °C), 130 μL of supernatant was mixed with 250 μL of ultrapure water and subjected to ultrafiltration using 3-kDa cutoff filters (Amicon Ultra 3 K device, Merck, Darmstadt, Germany), followed by 30 min of evaporation and 6 h of lyophilization. The lyophilized specimens were reconstituted in 200 μL of ultrapure water, further diluted if necessary, and then subjected to LC–MS/MS (LCMS-8030, Shimadzu, Kyoto, Japan) analysis, based on the Method Package for Primary Metabolites ver. 2 (Shimadzu), according to the manufacturer’s protocol.

### 2.4. Cell Culture and Cell Experiments

The Fao cells (rat hepatoma cell line, ECACC #EC89042701, Salisbury, UK) were grown in Ham’s F12 medium supplemented with 10% FBS and antibiotics. The HepG2 cells (human hepatoma cell line, ATCC HB-8065, Manassas, VA, USA) were grown in DMEM supplemented with 10% FBS and antibiotics. The rat primary hepatocytes were prepared as described in a previous report [14] and cultured in Williams’ E medium supplemented with 10% FBS and antibiotics. All cells were cultured in 5% CO_2_ at 37 °C.

When cells reached sub-confluency, the medium was changed to the experimental medium (Table S1), and the cells were cultured for the indicated durations. A concentrated Full medium was added to the culture medium to stimulate cells after amino acid starvation.

### 2.5. RNA Extraction and Real-Time Quantitative PCR (qPCR)

The total RNA was extracted from liver pieces or cells using TRIzol Reagent (Invitrogen, Carlsbad, CA, USA), and cDNA synthesis was carried out using the ReverTra Ace qPCR RT Master Mix (Toyobo, Osaka, Japan). The cDNA was subjected to qPCR using the THUNDERBIRD SYBR qPCR Mix (Toyobo) and Real-Time PCR system 7500 TH (Life Technologies, Carlsbad, CA, USA). For all samples, *Actb* mRNA was measured as an internal control and used for data normalization. The primer sets used are shown in Table S5.

### 2.6. Immunoblotting

Cell lysates were prepared using a standard method and were subjected to SDS-PAGE. Gel-separated proteins were transferred to PVDF membranes (Merck Millipore, Billerica, MA, USA), and protein bands were visualized using Western Lightning Plus-ECL, Enhanced Chemiluminescence Substrate (PerkinElmer, Waltham, MA, USA). The following primary antibodies were used: anti-GHR antibody (Abcam, Cambridge, England, #ab134078), anti-JAK2 antibody (Upstate, Darmstadt, Germany, #06-255), anti-phospho-JAK2 antibody (CST, Danvers, MA, USA, #3771), anti-STAT5b antibody (Abcam, Cambridge, UK, #ab178941), anti-phospho-STAT5a/b antibody (Santa Cruz Biotechnology, Santa Cruz, CA, USA, #sc-81524), anti-LC3 antibody (Norvus Bio, CO, USA, #NB-100-2220SS), anti-β-actin antibody (Sigma Aldrich, St. Louis, MO, USA #AC-15), and anti-HSP90α/β antibody (Santa Cruz, CA, USA #sc13119).

### 2.7. Statistical Analysis

Data are expressed as mean ± standard error of the mean (SEM). Comparisons between two groups were performed using Student’s *t*-test. Comparisons among more than two groups were performed using one-way or two-way analysis of variance (ANOVA). If the *p*-value obtained from the ANOVA was less than 0.05, post-hoc tests indicated in each figure legend were performed. Differences were considered statistically significant at *p* < 0.05. All statistical calculations were performed using JMP^®^ Pro (SAS Institute Inc., Cary, NC, USA).

## 3. Results

### 3.1. Dietary EAA Restriction Causes Serum IGF-I Reduction and Growth Retardation in Young Rats

To confirm the effect of dietary EAA deficiency on growth, we first prepared experimental diets containing an amino acid mixture as the sole nitrogen source (Table S1). The amino acid composition of the mixture was equivalent to that of casein plus methionine supplementation. Based on our previous study, we chose 15% of the total amino acid content as the control complete diet (CN diet) and 5% (each amino acid content was one-third of that of the CN diet) as the low amino acid diet (5AA diet) [14]. Diets deficient in a single amino acid were prepared in the same way as the CN diet, except that the content of single amino acid was one-third that of the CN diet (Δ-- diet). These diets were given to six-week-old young male Wistar rats for 7 d.

**Figure 1:**
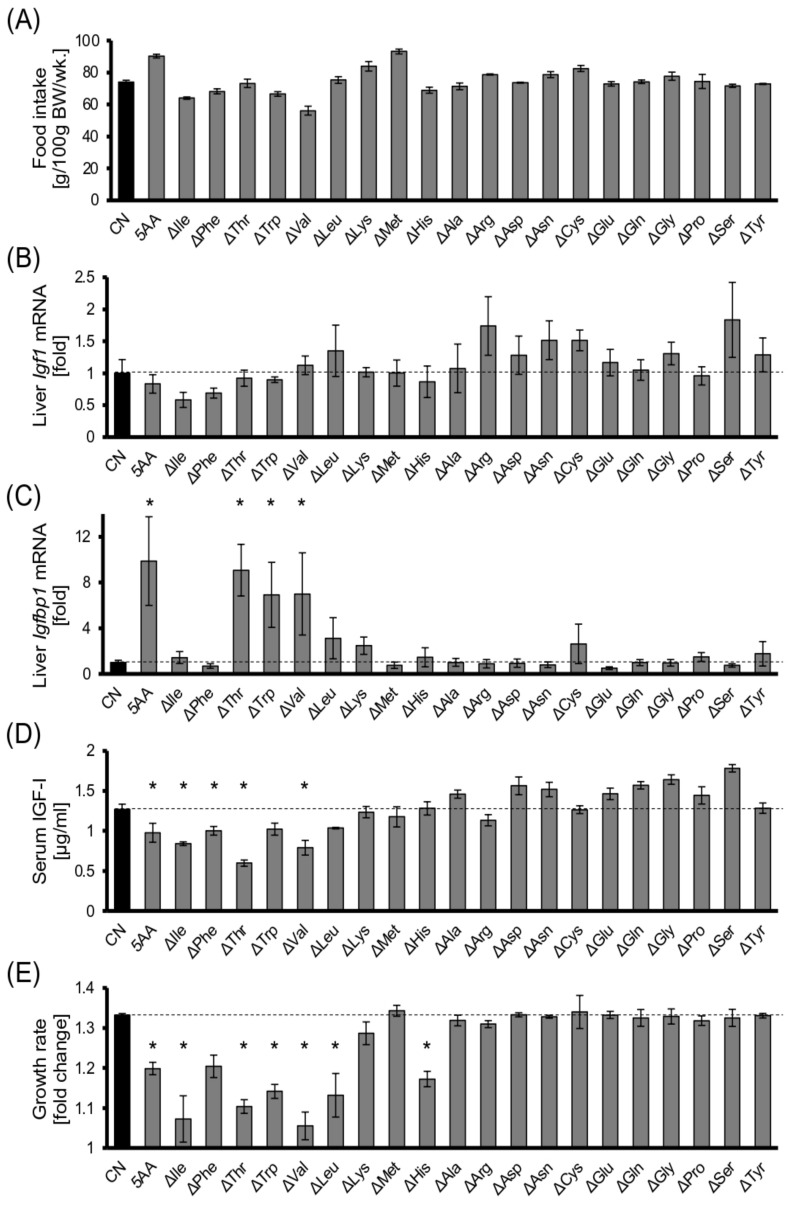
Essential amino acid intake is required to sustain serum IGF–I levels. Six-week-old male Wistar rats were reared on the control diet containing a 15% amino acid mixture as the sole nitrogen source (CN), a low–amino acid diet containing 5% amino acids (5AA), and diets where only a single amino acid was reduced to the same level as that of the 5AA diet (Δ---) for 7 days. (**A**),Total food intake during the experiment; (**B**,**C**) Liver *Igf1* and *Igfbp1* mRNA levels were measured using real–time qPCR. The values were normalized against 18S rRNA; (**D**) Serum IGF–I levels; (**E**) The ratio of the final body weight to the initial body weight; Bar: mean ± S.E.M., * *p* < 0.05, Dunnett’s test (vs. CN), *n* = 3.

Consistent with our previous study [14], the 5AA diet caused a significant reduction in serum IGF-I levels, followed by a smaller body weight gain, compared with that of CN diet-fed rats, although the 5AA group had a slightly larger amount of food intake (Figure 1). A tendency for decreased *Igf1* mRNA accompanied this and significantly increased *Igfbp1* mRNA levels in the 5AA livers (Figure 1). IGF binding protein 1 (IGFBP1) is a secreted protein, mainly produced by the liver, which interferes with IGF-I action by binding to it [15]. The ΔIle and ΔPhe diets tended to decrease the hepatic *Igf1* mRNA levels; ΔThr, ΔTrp, and ΔVal enhanced the *Igfbp1* mRNA levels; and ΔLeu and ΔLys tended to increase the *Igfbp1* mRNA in the liver, resulting in a reduction in the serum IGF-I level, except for ΔLys (Figure 1). These results were consistent with the bodyweight change; that is, growth retardation caused by EAA restriction correlated well with the decrease in serum IGF-I levels with one exception, namely, ΔHis. The mRNA levels of another major IGF binding protein, Igfbp3, were slightly, but not statistically significantly, different among the experimental groups and showed no marked correlation with serum IGF-I concentrations or growth rate (Figure S1).

### 3.2. IGF-I Infusion Restores the Growth Retardation under Protein Malnutrition

**Figure 2:**
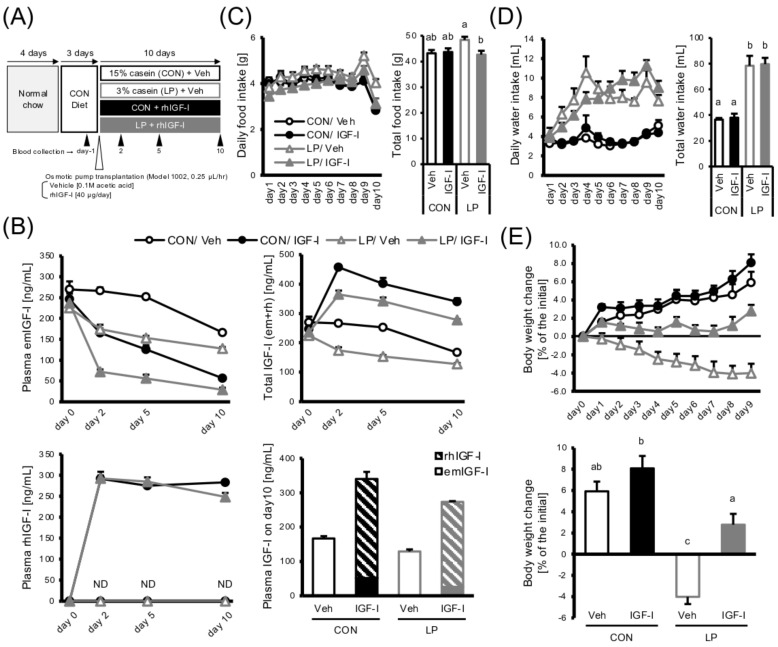
IGF–I infusion restores growth retardation caused by dietary amino acid deficiency. (**A**) Schematic of the experimental design. Seven-week-old male C57BL/6 mice were fed standard chow for 4 days, followed by a 15% casein experimental diet (CON) for 3 days, for training. Then, osmotic pumps carrying either rhIGF–I or vehicle were transplanted into the mice, and they were fed either a CON diet or 3% casein low–protein diet (LP) for another 10 days; (**B**) Plasma IGF–I concentration was measured on days 0, 2, 5, and 10. Endogenous emIGF–I (**upper left**) and exogenous rhIGF–I (**lower left**) of each sample were separately analyzed, and total plasma IGF–I was calculated as the sum of both (**upper right**). The total plasma IGF–I concentration on the last day is also indicated by a bar graph; (**C**), Daily food intake (**left**), and total food intake during the experimental period (**right**). (**D**) Daily water intake (**left**) and total water intake during the experimental period (**right**). (**E**) The bodyweight change of each mouse is indicated as a percent increase (or decrease) of the initial body weight (**upper**). The ratio of the final body weight to the initial body weight is also indicated as a bar graph (**lower**). Statistically significant differences were observed between groups with different alphabetical characters; Bar: mean ± S.E.M. (**C**–**E**): *n* = 6, Tukey–Kramer test; (**B**): *n* = 3–6, Tukey–Kramer test. ND means not detected.

To determine whether the downregulation of serum IGF-I levels upon amino acid-deficient diet feeding was the cause of growth retardation, a rescue experiment was performed by administering recombinant human IGF-I (rhIGF-I). Either a control diet (CON; containing 15% casein as a nitrogen source) or a low-protein diet (LP; 3% casein) (Table S2) was given to eight-week-old male mice that were implanted with an osmotic pump releasing either rhIGF-I or vehicle for 10 d (Figure 2A). In both diet groups, rhIGF-I infusion significantly decreased the endogenous mouse IGF-I (emIGF-I) levels (Figure 2B). However, the total circulating IGF-I (rhIGF-I + emIGF-I) was much higher in rhIGF-I-treated mice than in vehicle-treated animals (emIGF-I only), ensuring that reduced serum IGF-I levels caused by LP feeding were successfully rescued.

Similar to those in previous studies reported [16], LP feeding moderately increased food intake. Still, IGF-I-infusion normalized it, making the food intake of CON- and LP-fed mice comparable, while drinking was not affected (Figure 2C,D). Remarkably, while the mice fed the LP diet lost their weight by ~5% during the experimental period, the IGF-I load caused a significant recovery in the body weight gain, even though their diet was still deficient in amino acids and their requirements were not satisfied (Figure 2E, Table S3). Autophagy activity in the skeletal muscle was slightly enhanced by IGF-I infusion, as indicated by the protein amount of the lipidated form of LC3 (LC3-II). In contrast, the 3-methylhistidine concentration in the plasma did not change, suggesting that skeletal muscle protein breakdown was almost comparable (Figure S1). These results challenge the conventional idea that animals will not grow unless EAA requirements are met fully to supply building blocks, as they cannot be synthesized in the body.

### 3.3. EAAs Are Required for Igf1 Transcription in Hepatocyte Models

**Figure 3:**
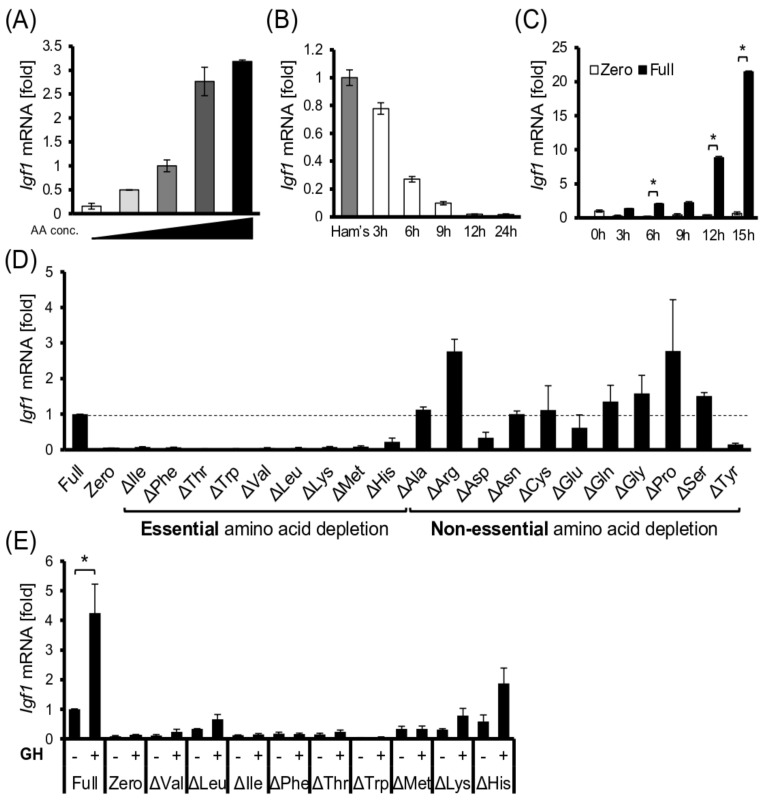
Essential amino acids are required for *Igf1* mRNA expression; (**A**) Fao cells were cultured in the Full medium and media wherein the amino acid content was changed by 3, 2, 0.5, and 0 (Zero) times for 24 h, and then *Igf1* mRNA levels were quantified using real–time qPCR; (**B**) Fao cells were cultured in Zero medium for 3, 6, 9, 12, and 24 h, and *Igf1* mRNA levels were quantified using real–time qPCR; (**C**) Fao cells were amino acid-starved by pre-culturing for 9 h in Zero medium. Then, a full or zero medium was added for stimulation, and cells were cultured for another 3, 6, 9, 12, and 15 h. *Igf1* mRNA levels were quantified using real–time qPCR; (**D**) Fao cells were cultured in Full, Zero, or Full medium lacking a single amino acid (Δ---) for 24 h. Then, *Igf1* mRNA levels were quantified using real–time qPCR; (**E**) Fao cells were cultured in the indicated medium with or without 100 nM GH for 24 h. Then, *Igf1* mRNA levels were quantified using real–time qPCR. Data information: *Igf1* mRNA levels were normalized against *Actb* mRNA levels. Bar graphs are presented as the fold change of columns on the far left or Full. Bar: mean ± S.E.M., * *p* < 0.05, Student’s *t*-test, *n* = 3.

Accumulating evidence has shown that the transcription of several metabolic genes in hepatocytes is regulated in response to changes in extracellular amino acid concentration in a cell-autonomous manner [17,18]. In addition, the concentration of most EAAs in the serum was significantly decreased by feeding diets deficient in the corresponding EAAs (Figure S2). Therefore, we tested the response of cultured hepatocyte models to amino acids in the media. When Fao rat hepatoma cells, human HepG2 hepatoma cells, and rat primary hepatocytes were cultured in serum-free complete medium (Full medium) or amino acid-depleted medium (Zero medium) for 24 h, *Igf1* mRNA expression was much lower in the Zero medium (Table S4, Figure 3A and Figure S3). As the medium amino acid concentrations varied from zero to three times that of the Full medium, *Igf1* mRNA levels in the cells increased proportionally to those of the surrounding amino acid levels (Figure 3A). *Igf1* mRNA levels in the cells gradually declined after changing their media to Zero, and when amino acids were re-added after amino acid starvation, the mRNA levels increased significantly again (Figure 3B,C). These results demonstrated that liver-derived cells reversibly changed the *Igf1* mRNA levels, corresponding to extracellular amino acid levels without any endocrine signals or neuronal input.

We then assessed the contribution of each amino acid in the cell culture model. When any of the EAAs in the Full medium was depleted, *Igf1* mRNA levels in the Fao cells were considerably downregulated compared with those in the complete medium (Figure 3D). In contrast, when the cells were cultured in the non-essential amino acid (NEAA)-deprived medium, *Igf1* mRNA levels did not change or even slightly increased in most cases, except for ΔAsp and ΔTyr medium cultures displaying reduced *Igf1* mRNA (Figure 3D). These results suggest that EAAs are required for cellular protein metabolism and *Igf1* mRNA levels.

### 3.4. EAA Deprivation Induces GH Resistance in the Hepatocyte Model

**Figure 4:**
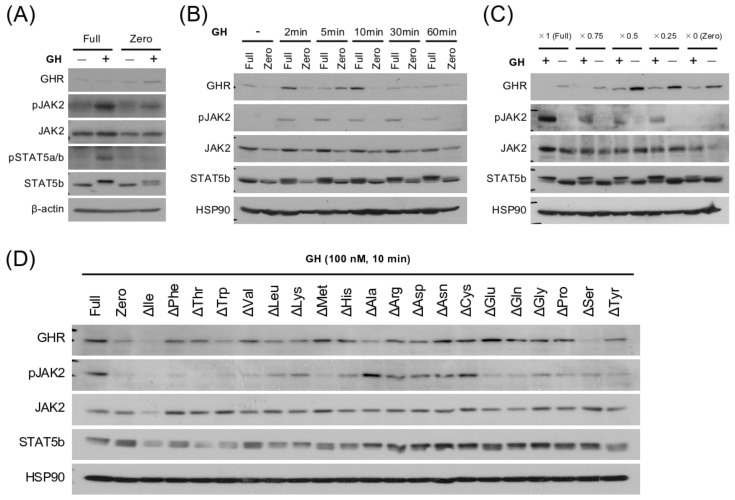
Essential amino acids are required for GH signal transduction; (**A**) Fao cells were cultured in Full or Zero medium for 24 h, and 100 nM GH was added. After 10 min of GH stimulation, proteins related to GH signaling were analyzed by immunoblotting; (**B**) Fao cells were cultured in Full or Zero medium for 24 h, and then 100 nM GH was added, and cells were incubated for another 2, 5, 10, 30, and 60 min. Proteins related to GH signaling were analyzed by immunoblotting.(**C**) Fao cells were cultured in Full medium and media with amino acid concentrations of 0.75, 0.5, 0.25, and 0 (Zero) times. Full medium concentration for 24 h; 100 nM GH was added. After 10 min of GH stimulation, proteins related to GH signaling were analyzed by immunoblotting. (**D**) Fao cells were cultured in Full, Zero, or Full medium lacking a single amino acid (Δ---) for 24 h, and then 100 nM GH was added. After 10 min of GH stimulation, proteins related to GH signaling were analyzed by immunoblotting.

Growth hormone is a major upstream regulator of liver IGF-I expression. Therefore, we investigated whether amino acids affected GH signaling. When Fao cells cultured in Full medium were treated with GH, the *Igf1* mRNA levels were significantly increased. However, the Fao cells did not respond to GH in EAA-deprived media, and *Igf1* mRNA did not increase (Figure 3E). Consistently, phosphorylation of JAK2 and STAT5 upon GH stimulation was markedly inhibited by amino acid starvation (Figure 4A–C). Furthermore, depletion of any EAAs in the Full medium decreased the GH-induced phosphorylation of JAK2 and STAT5, while NEAA depletion did not (Figure 4D). These results suggest that GH signal transduction requires medium EAAs to regulate *Igf1* transcription in the hepatocyte model. Only a single EAA deprivation was sufficient to intercept the GH signaling almost completely, illustrating that EAAs per se are involved in the mechanisms of *Igf1* transcriptional regulation and also that they are the upstream modulators of the GH/IGF-I axis.

## 4. Discussion

It has long been believed that EAAs should be included in a diet to promote body growth because they are not synthesized in the animal body. This concept led to the idea of “minimal amino acid requirement,” and many researchers have determined that of laboratory animals and humans, based on the concept [19,20]. Looking into those references, the protein requirements of young rats and mice for optimal growth are ~10% to ~15% and ~15% to ~25%, respectively. The low-protein diets we used (5% and 3% protein) were short of protein. Furthermore, the content of all EAAs in the diets was also much lower than their requirements by less than half [19]. In addition, rats and mice that were fed these diets actually showed an attenuated body weight increase, suggesting that their EAA requirements were not fulfilled. However, under the same conditions, IGF-I infusion successfully rescued the body weight gain (Figure 2E). This interesting result could be controversial considering the conventional concept that if animals are prevented from sufficient EAA intake, they cannot grow. Only in the presence of sufficient IGF-I in the circulation can animals potentially grow even under EAA-deficient conditions, at least within a short period (~two weeks). Indeed, the animals cannot produce sufficient EAAs by themselves. If the EAA deficiency is prolonged for more than an acceptable duration, the shortage may inevitably become serious, possibly resulting in growth failure and death.

We previously demonstrated that the liver is sensitive to the protein nutritional status of the organism, and its metabolism is dynamically regulated to adapt to the environment [14,17,18,21]. Similarly, a low-EAA diet significantly decreased the serum concentration of the corresponding EAA, which strikingly affected the *Igf1* and *Igfbp1* transcription in the liver, resulting in a marked reduction in circulating IGF-I levels (Figure 1). Consistently, depriving the culture medium of EAAs decreased the *Igf1* transcription in the Fao cells (Figure 3A–D). These results suggest a cell-autonomous mechanism independent of GH in hepatic *Igf1* transcriptional regulation in response to extracellular amino acids. Meanwhile, amino acid depletion also diminished the activation of the JAK/STAT pathway upon GH stimulation (Figure 4A–D). In summary, hepatic IGF-I production is regulated directly and indirectly by amino acids, enabling the animals to adapt their body size corresponding to the nutritional environment.

Why was IGF-I administration able to promote body growth, even during the EAA intake was insufficient? Under EAA-deficient conditions, serum EAA levels decrease, and autophagy activity in each tissue can be potentiated (Figures S1 and S2) [22]. This may facilitate the recycling of EAAs systemically, allowing tissues to tolerate acute EAA deficiency. In addition, the gut microbiome can synthesize EAAs de novo and supply them to the host, which may also provide a minimum of the necessary EAAs [23]. Dietary EAA restriction normally impairs serum IGF-I activity, which causes growth retardation, presumably to save limited resources. However, owing to the remaining minimal supply of EAAs, the animal may still have the potential ability to grow for a while if a sufficient dose of IGF-I is available. Thus, overall, IGF-I administration promoted body growth, even under the EAA-insufficient conditions.

It should be noted that there are several reports of IGF-I infusion experiments around 1990, and all of them mention the failure of IGF-I infusion to promote the growth of the malnourished animals [24,25]. The difference between these reports and the present study is that the previous studies used: (1) rats of (2) much younger age, (3) females, (4) different diets (i.e., diets containing protein (e.g., casein) as a nitrogen source, not AA mixture), and (5) different methodologies (i.e., subcutaneous injection every day). The minimum protein/EAA requirement and the efficacy of protein/EAA usage are also different between mice and rats. It has been reported that dietary protein availability affects the GH/IGF-I resistance in animals [26]. Induction of GH resistance by EAA deprivation was also observed in our model (Figure 3E and Figure 4). These differences might account for our finding that IGF-I could promote growth under a low EAA regime. Furthermore, another striking issue is that Yakar et al., clearly showed that the loss of serum IGF-I was less involved in body growth [27]. They generated liver-specific IGF-I knockout mice, which exhibited a nearly 75% reduction in serum IGF-I, but there were no postnatal developmental defects. These results suggest that endocrine IGF-I and local IGF-I action in each tissue is significant. From that perspective, a low-protein diet or amino acid deficiency enhances insulin sensitivity. It potentiates insulin-like signaling both in vivo and cultured hepatocytes [14,28], implying that animals with protein malnutrition can be sensitive to insulin-like peptides in certain cases. Thus, the overload of exogenous IGF-I succeeded in recovering growth in the current study by unknown mechanisms.

In yeasts, nutrients, including amino acids, may become direct signals to regulate metabolism. In *C. elegans* and *Drosophila*, environmental changes, including nutrient status, are monitored by a series of insulin-like peptides which are secreted from the central nervous system. These information sources converge onto one insulin-like peptide receptor and subsequent conventional signaling pathways. However, in fish and mammals, insulin-like peptides are produced in various peripheral tissues. Their synthesis and levels are regulated by factors such as the central nervous system, hypothalamus-pituitary endocrine system, and IGF binding proteins that respond to environmental changes [29]. In this evolutionary context, it is probable that the amino acid signaling system controlling IGF-I production exists before the development of the GH/IGF-I system, illustrating how prominent the effects of amino acids on IGF-I synthesis are also in mammals, even in mammals in the presence of GH.

In summary, we demonstrated that protein/EAA deficiency inhibited IGF-I production in two ways: directly acting on *Igf1* transcription and indirectly interfering with the *Igf1* transcription by interrupting GH signaling, leading to eventual growth retardation. Restoration of IGF-I successfully rescued the defect, even though protein malnutrition was still ongoing. These results suggest a new paradigm of EAA that its intake from a diet is not necessarily required for body growth but is essential as an IGF-I-tropic cue for the proper maintenance of the GH/IGF-I axis and the facilitation of IGF-I action. Children with kwashiorkor, a severe condition resulting from inadequate protein intake, exhibit low serum GH and IGF-I levels, growth retardation, and GH resistance [30]. This implies a possible application of the mechanism in question in human nutrition or as a potential clinical treatment for growth disorders.

## Data Availability

The data presented in this study are available on request from the corresponding author.

## Supplementary Materials

The following supporting information can be downloaded at: https://www.mdpi.com/article/10.3390/cells11091523/s1, Figure S1: Igfbp3 mRNA levels of the rat liver, related to Figure 1; Figure S2: Indicators of the skeletal muscle protein breakdown, related to Figure 2; Figure S3: Rat serum amino acid concentration, related to Figure 1; Figure S4: Amino acids are required for IGF-I mRNA expression in cultured hepatocyte models, related to Figure 3; Table S1: Amino acid composition of the experimental diets, related to Figure 1; Table S2: Composition of experimental diets, related to Figure 2; Table S3: Tissue weight of mice, related to Figure 2; Table S4: Amino acid composition of the experimental media, related to Figure 3; Table S5: Primer list.
