# Supplementary material for: Essential Amino Acid Intake Is Required for Sustaining Serum Insulin-like Growth Factor-I Levels but Is Not Necessarily Needed for Body Growth
Source: Cells. 2022 May 2;11(9):1523. doi: 10.3390/cells11091523 (PMC9105520; doi:10.3390/cells11091523)
Supplement: Supplementary file 1 [file cells-11-01523-s001.zip › cells-1674197-supplementary.pptx]

## Slide 1
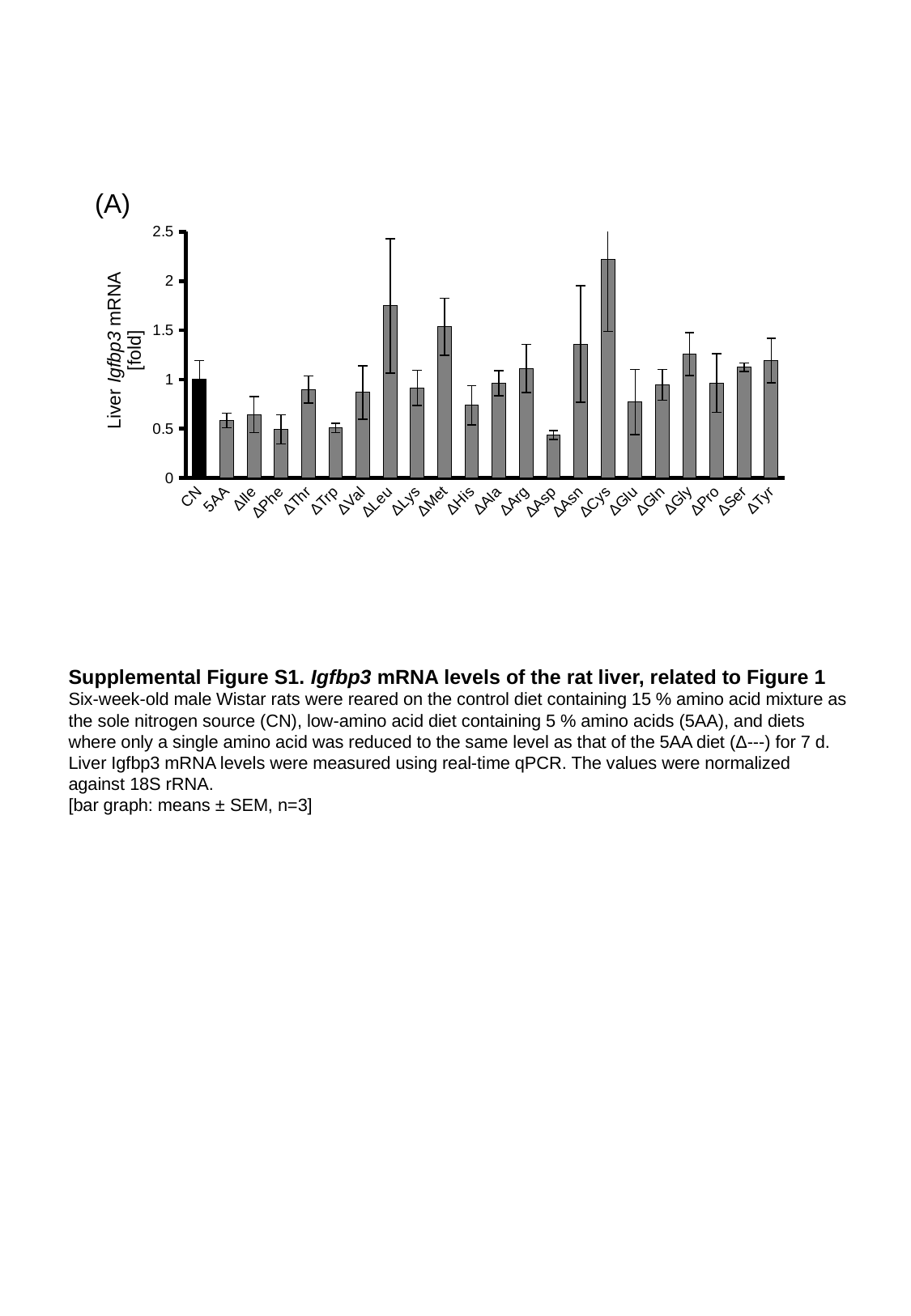

(A)
### Chart
| Category | |
|---|---|
| CN | 1.0 |
| 5AA | 0.5858499887108392 |
| ΔIle | 0.6441100063053811 |
| ΔPhe | 0.4944115851549364 |
| ΔThr | 0.9002799805787333 |
| ΔTrp | 0.5088654279563738 |
| ΔVal | 0.8689204541903385 |
| ΔLeu | 1.7476925567398485 |
| ΔLys | 0.916586585894322 |
| ΔMet | 1.5359253882584547 |
| ΔHis | 0.7368477043273453 |
| ΔAla | 0.9613714810151476 |
| ΔArg | 1.1124975187130222 |
| ΔAsp | 0.4357677265240241 |
| ΔAsn | 1.3601222251342744 |
| ΔCys | 2.2227292682400006 |
| ΔGlu | 0.7716848595043032 |
| ΔGln | 0.946003338672344 |
| ΔGly | 1.2577069575939777 |
| ΔPro | 0.9641912668480824 |
| ΔSer | 1.1253627405225481 |
| ΔTyr | 1.1913125406337939 |Liver Igfbp3 mRNA
[fold]
Supplemental Figure S1. Igfbp3 mRNA levels of the rat liver, related to Figure 1
Six-week-old male Wistar rats were reared on the control diet containing 15 % amino acid mixture as the sole nitrogen source (CN), low-amino acid diet containing 5 % amino acids (5AA), and diets where only a single amino acid was reduced to the same level as that of the 5AA diet (Δ---) for 7 d.
Liver Igfbp3 mRNA levels were measured using real-time qPCR. The values were normalized against 18S rRNA.
[bar graph: means ± SEM, n=3]

## Slide 2
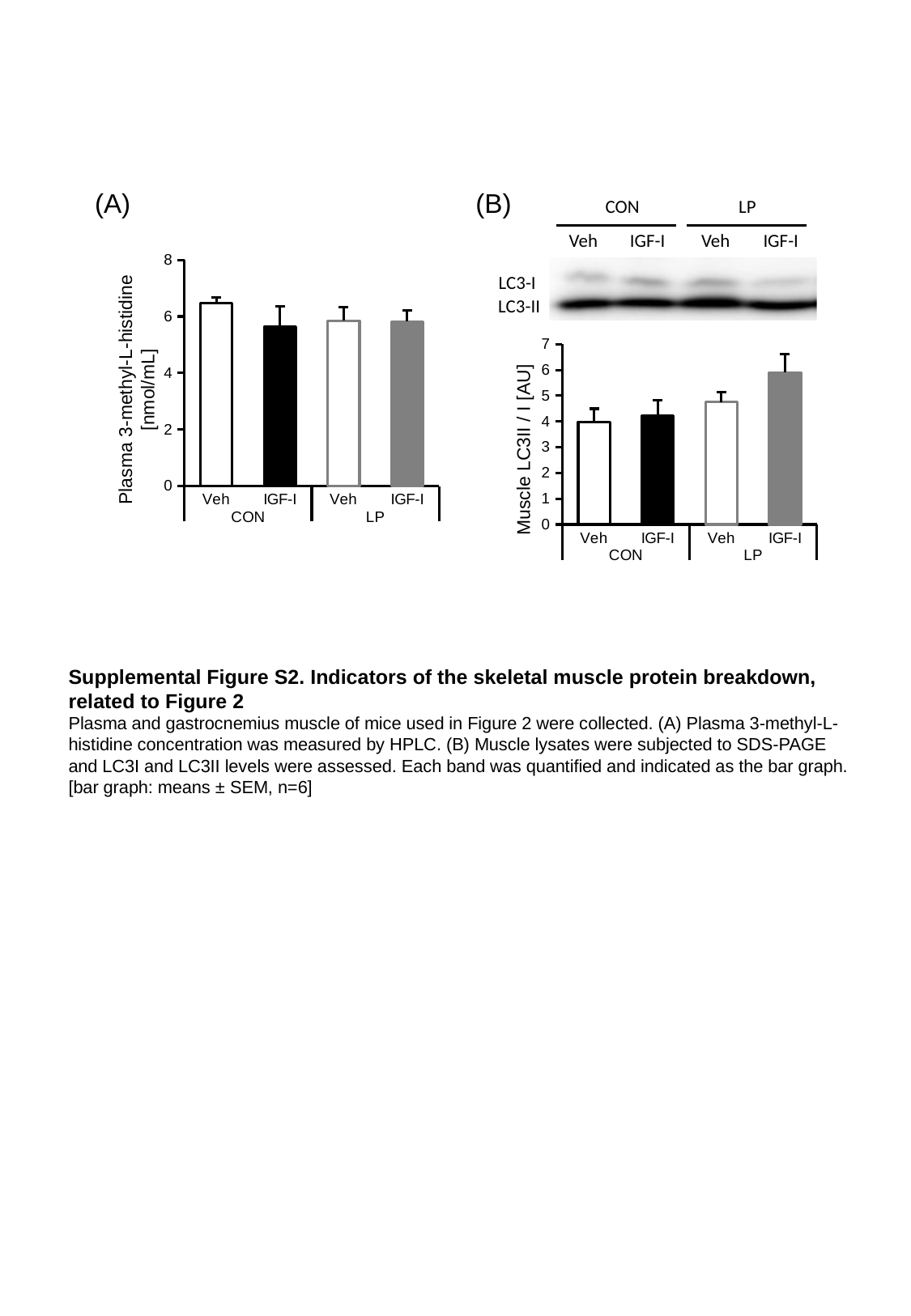

(A)
(B)
CON
LP
Veh
IGF-I
Veh
IGF-I
### Chart
| Category | |
|---|---|
| Veh | 6.470677182781041 |
| IGF-I | 5.6274285166720155 |
| Veh | 5.847803163856039 |
| IGF-I | 5.8217414533780785 |
LC3-I
LC3-II
### Chart
| Category | |
|---|---|
| Veh | 3.9802467054153725 |
| IGF-I | 4.242236692117282 |
| Veh | 4.759531976836975 |
| IGF-I | 5.894969541897401 |Plasma 3-methyl-L-histidine
[nmol/mL]
Muscle LC3II / I [AU]
Supplemental Figure S2. Indicators of the skeletal muscle protein breakdown, related to Figure 2
Plasma and gastrocnemius muscle of mice used in Figure 2 were collected. (A) Plasma 3-methyl-L-histidine concentration was measured by HPLC. (B) Muscle lysates were subjected to SDS-PAGE and LC3I and LC3II levels were assessed. Each band was quantified and indicated as the bar graph.
[bar graph: means ± SEM, n=6]

## Slide 3
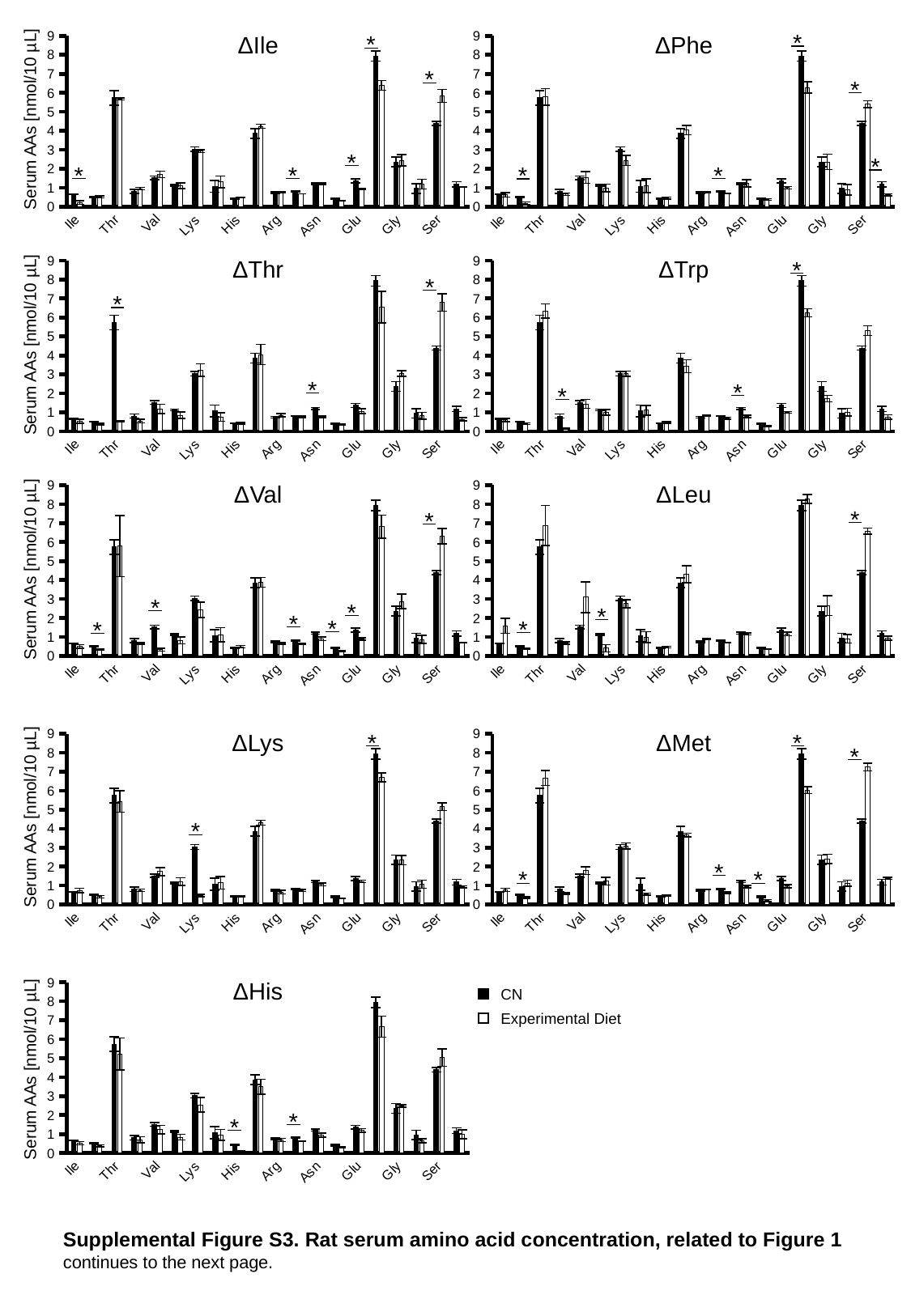

*
*
### Chart
| Category | | |
|---|---|---|
| Ile | 0.6629947811983596 | 0.22207596532922422 |
| Phe | 0.5127424672643781 | 0.5134616627371178 |
| Thr | 5.740336254712299 | 5.676464036648492 |
| Trp | 0.8058057282365031 | 0.9531961487615774 |
| Val | 1.516330666114073 | 1.6926784430359942 |
| Leu | 1.1274763637164218 | 1.100194912111105 |
| Lys | 3.030054349987205 | 2.929408987978896 |
| Met | 1.0717299640812552 | 1.3093086308278987 |
| His | 0.42909038293339835 | 0.48209953256535215 |
| Ala | 3.86070659483901 | 4.2392154873114665 |
| Arg | 0.7457233480199355 | 0.7686346051900405 |
| Asp | 0.8085383792348607 | 0.6784851082908374 |
| Asn | 1.208845890511536 | 1.20752646704751 |
| Cys | 0.40998024346180045 | 0.31857819965097217 |
| Glu | 1.3633755770996656 | 0.9172892667548945 |
| Gln | 7.9374491586874 | 6.388619159655679 |
| Gly | 2.358812546598679 | 2.4417850503475513 |
| Pro | 0.9548825123662348 | 1.188208721813937 |
| Ser | 4.389129553042212 | 5.837746622078664 |
| Tyr | 1.1781332174282957 | 1.0257706211102156 |ΔIle
### Chart
| Category | | |
|---|---|---|
| Ile | 0.6629947811983596 | 0.6255465581979219 |
| Phe | 0.5127424672643781 | 0.18332668891948178 |
| Thr | 5.740336254712299 | 5.7839251390389075 |
| Trp | 0.8058057282365031 | 0.6579544103976692 |
| Val | 1.516330666114073 | 1.5360380901501969 |
| Leu | 1.1274763637164218 | 0.9874196534065103 |
| Lys | 3.030054349987205 | 2.4396472640164055 |
| Met | 1.0717299640812552 | 1.1043828166132437 |
| His | 0.42909038293339835 | 0.45734006323628335 |
| Ala | 3.86070659483901 | 4.035950104519775 |
| Arg | 0.7457233480199355 | 0.7475060200594794 |
| Asp | 0.8085383792348607 | 0.6894531621936081 |
| Asn | 1.208845890511536 | 1.2252987970621967 |
| Cys | 0.40998024346180045 | 0.38409215489507265 |
| Glu | 1.3633755770996656 | 0.9972860531487419 |
| Gln | 7.9374491586874 | 6.286105251210796 |
| Gly | 2.358812546598679 | 2.356147933397326 |
| Pro | 0.9548825123662348 | 0.8964700647190634 |
| Ser | 4.389129553042212 | 5.394553101109864 |
| Tyr | 1.1781332174282957 | 0.6209207608748412 |ΔPhe
*
*
Serum AAs [nmol/10 µL]
*
*
*
*
*
*
### Chart
| Category | | |
|---|---|---|
| Ile | 0.6629947811983596 | 0.526677478395866 |
| Phe | 0.5127424672643781 | 0.38888092385614675 |
| Thr | 5.740336254712299 | 0.5421185622004433 |
| Trp | 0.8058057282365031 | 0.5444657986620379 |
| Val | 1.516330666114073 | 1.1716918271022168 |
| Leu | 1.1274763637164218 | 0.8487778681069362 |
| Lys | 3.030054349987205 | 3.223840807174233 |
| Met | 1.0717299640812552 | 0.7517787509630213 |
| His | 0.42909038293339835 | 0.4179318541146189 |
| Ala | 3.86070659483901 | 4.049595173629415 |
| Arg | 0.7457233480199355 | 0.8451097343672923 |
| Asp | 0.8085383792348607 | 0.7679942932282855 |
| Asn | 1.208845890511536 | 0.77032196917906 |
| Cys | 0.40998024346180045 | 0.36999357577630637 |
| Glu | 1.3633755770996656 | 1.0555679784531682 |
| Gln | 7.9374491586874 | 6.5387468060131715 |
| Gly | 2.358812546598679 | 3.052369739278482 |
| Pro | 0.9548825123662348 | 0.8250049250716088 |
| Ser | 4.389129553042212 | 6.793215504173166 |
| Tyr | 1.1781332174282957 | 0.6362349069636849 |ΔThr
### Chart
| Category | | |
|---|---|---|
| Ile | 0.6629947811983596 | 0.5972352694831717 |
| Phe | 0.5127424672643781 | 0.4082386797836873 |
| Thr | 5.740336254712299 | 6.338375767873899 |
| Trp | 0.8058057282365031 | 0.14143926080554914 |
| Val | 1.516330666114073 | 1.444922737358458 |
| Leu | 1.1274763637164218 | 1.0008595063605596 |
| Lys | 3.030054349987205 | 3.0368039179548094 |
| Met | 1.0717299640812552 | 1.1075508016878042 |
| His | 0.42909038293339835 | 0.4653379877327852 |
| Ala | 3.86070659483901 | 3.430966961929416 |
| Arg | 0.7457233480199355 | 0.8186313299130407 |
| Asp | 0.8085383792348607 | 0.693436139062475 |
| Asn | 1.208845890511536 | 0.7828162124211479 |
| Cys | 0.40998024346180045 | 0.26836713971076154 |
| Glu | 1.3633755770996656 | 0.9970556354893038 |
| Gln | 7.9374491586874 | 6.245678405889372 |
| Gly | 2.358812546598679 | 1.7262672655443074 |
| Pro | 0.9548825123662348 | 1.0067959734383025 |
| Ser | 4.389129553042212 | 5.307647174440078 |
| Tyr | 1.1781332174282957 | 0.756170136120987 |ΔTrp
*
*
*
Serum AAs [nmol/10 µL]
*
*
*
### Chart
| Category | | |
|---|---|---|
| Ile | 0.6629947811983596 | 0.4947189754004199 |
| Phe | 0.5127424672643781 | 0.35235549255987975 |
| Thr | 5.740336254712299 | 5.790612492957179 |
| Trp | 0.8058057282365031 | 0.6656213614014858 |
| Val | 1.516330666114073 | 0.3293418130629662 |
| Leu | 1.1274763637164218 | 0.8127282897174918 |
| Lys | 3.030054349987205 | 2.4290636366687632 |
| Met | 1.0717299640812552 | 1.1212656421472478 |
| His | 0.42909038293339835 | 0.48293051901312395 |
| Ala | 3.86070659483901 | 3.8846504109720663 |
| Arg | 0.7457233480199355 | 0.6597658144578443 |
| Asp | 0.8085383792348607 | 0.6299137021980011 |
| Asn | 1.208845890511536 | 0.8988697587957356 |
| Cys | 0.40998024346180045 | 0.2632283622732664 |
| Glu | 1.3633755770996656 | 0.8919941013362166 |
| Gln | 7.9374491586874 | 6.8144301190882075 |
| Gly | 2.358812546598679 | 2.8738433782780564 |
| Pro | 0.9548825123662348 | 0.8728063563496621 |
| Ser | 4.389129553042212 | 6.315210508496825 |
| Tyr | 1.1781332174282957 | 0.7140571294823359 |ΔVal
### Chart
| Category | | |
|---|---|---|
| Ile | 0.6629947811983596 | 1.5932388290025814 |
| Phe | 0.5127424672643781 | 0.38707091332018484 |
| Thr | 5.740336254712299 | 6.870513008658469 |
| Trp | 0.8058057282365031 | 0.6851288806373276 |
| Val | 1.516330666114073 | 3.0918199994725355 |
| Leu | 1.1274763637164218 | 0.40569484981960663 |
| Lys | 3.030054349987205 | 2.7547485401787903 |
| Met | 1.0717299640812552 | 0.9931892135950844 |
| His | 0.42909038293339835 | 0.46653984665175674 |
| Ala | 3.86070659483901 | 4.306758437935033 |
| Arg | 0.7457233480199355 | 0.884364102523377 |
| Asp | 0.8085383792348607 | 0.6944143788272346 |
| Asn | 1.208845890511536 | 1.166836585665264 |
| Cys | 0.40998024346180045 | 0.3508389616486038 |
| Glu | 1.3633755770996656 | 1.1545621390306238 |
| Gln | 7.9374491586874 | 8.274136948326474 |
| Gly | 2.358812546598679 | 2.6449184755074584 |
| Pro | 0.9548825123662348 | 0.9033210280009651 |
| Ser | 4.389129553042212 | 6.577067160442428 |
| Tyr | 1.1781332174282957 | 0.9308484448257378 |ΔLeu
*
*
Serum AAs [nmol/10 µL]
*
*
*
*
*
*
*
### Chart
| Category | | |
|---|---|---|
| Ile | 0.6629947811983596 | 0.7349754268387757 |
| Phe | 0.5127424672643781 | 0.4089076750252534 |
| Thr | 5.740336254712299 | 5.429436006906119 |
| Trp | 0.8058057282365031 | 0.7481173097466184 |
| Val | 1.516330666114073 | 1.7314966027244656 |
| Leu | 1.1274763637164218 | 1.2028511837510258 |
| Lys | 3.030054349987205 | 0.471015813901378 |
| Met | 1.0717299640812552 | 1.138186683386951 |
| His | 0.42909038293339835 | 0.43756411755624763 |
| Ala | 3.86070659483901 | 4.3090790380065105 |
| Arg | 0.7457233480199355 | 0.6522544794967274 |
| Asp | 0.8085383792348607 | 0.7403992094878902 |
| Asn | 1.208845890511536 | 1.0628192506613896 |
| Cys | 0.40998024346180045 | 0.3331550313884477 |
| Glu | 1.3633755770996656 | 1.2169132045226878 |
| Gln | 7.9374491586874 | 6.7086710105849905 |
| Gly | 2.358812546598679 | 2.3320673521011948 |
| Pro | 0.9548825123662348 | 1.0675602256022634 |
| Ser | 4.389129553042212 | 5.148910138768226 |
| Tyr | 1.1781332174282957 | 0.9305481811479557 |ΔLys
### Chart
| Category | | |
|---|---|---|
| Ile | 0.6629947811983596 | 0.7710505153386573 |
| Phe | 0.5127424672643781 | 0.3587753137797967 |
| Thr | 5.740336254712299 | 6.667150855229572 |
| Trp | 0.8058057282365031 | 0.5836118163923794 |
| Val | 1.516330666114073 | 1.7871916900794513 |
| Leu | 1.1274763637164218 | 1.2324133103708539 |
| Lys | 3.030054349987205 | 3.083019802351989 |
| Met | 1.0717299640812552 | 0.5398198374302385 |
| His | 0.42909038293339835 | 0.4606550884550677 |
| Ala | 3.86070659483901 | 3.661570857792599 |
| Arg | 0.7457233480199355 | 0.8031509787998187 |
| Asp | 0.8085383792348607 | 0.6238111163505115 |
| Asn | 1.208845890511536 | 0.9454893821552887 |
| Cys | 0.40998024346180045 | 0.2044001358941877 |
| Glu | 1.3633755770996656 | 0.9673247459709629 |
| Gln | 7.9374491586874 | 6.033012869806067 |
| Gly | 2.358812546598679 | 2.4068568747788333 |
| Pro | 0.9548825123662348 | 1.1206749540570575 |
| Ser | 4.389129553042212 | 7.2419862824069385 |
| Tyr | 1.1781332174282957 | 1.3748051831854928 |ΔMet
*
*
*
Serum AAs [nmol/10 µL]
*
*
*
*
### Chart
| Category | | |
|---|---|---|
| Ile | 0.6629947811983596 | 0.5236063781125732 |
| Phe | 0.5127424672643781 | 0.38031937721380077 |
| Thr | 5.740336254712299 | 5.2303888376597385 |
| Trp | 0.8058057282365031 | 0.6935943845368024 |
| Val | 1.516330666114073 | 1.225534069388716 |
| Leu | 1.1274763637164218 | 0.8335710130884983 |
| Lys | 3.030054349987205 | 2.539349523306543 |
| Met | 1.0717299640812552 | 0.9573947583001443 |
| His | 0.42909038293339835 | 0.09828471868041823 |
| Ala | 3.86070659483901 | 3.498090537424696 |
| Arg | 0.7457233480199355 | 0.6918721243296554 |
| Asp | 0.8085383792348607 | 0.6291368165750478 |
| Asn | 1.208845890511536 | 0.9410592886240129 |
| Cys | 0.40998024346180045 | 0.315030088372501 |
| Glu | 1.3633755770996656 | 1.1896988328064888 |
| Gln | 7.9374491586874 | 6.664285235622221 |
| Gly | 2.358812546598679 | 2.48844207255449 |
| Pro | 0.9548825123662348 | 0.6559823609701954 |
| Ser | 4.389129553042212 | 5.028284799118109 |
| Tyr | 1.1781332174282957 | 0.9968869221627311 |ΔHis
CN
Experimental Diet
Serum AAs [nmol/10 µL]
*
*
Supplemental Figure S3. Rat serum amino acid concentration, related to Figure 1
continues to the next page.

## Slide 4
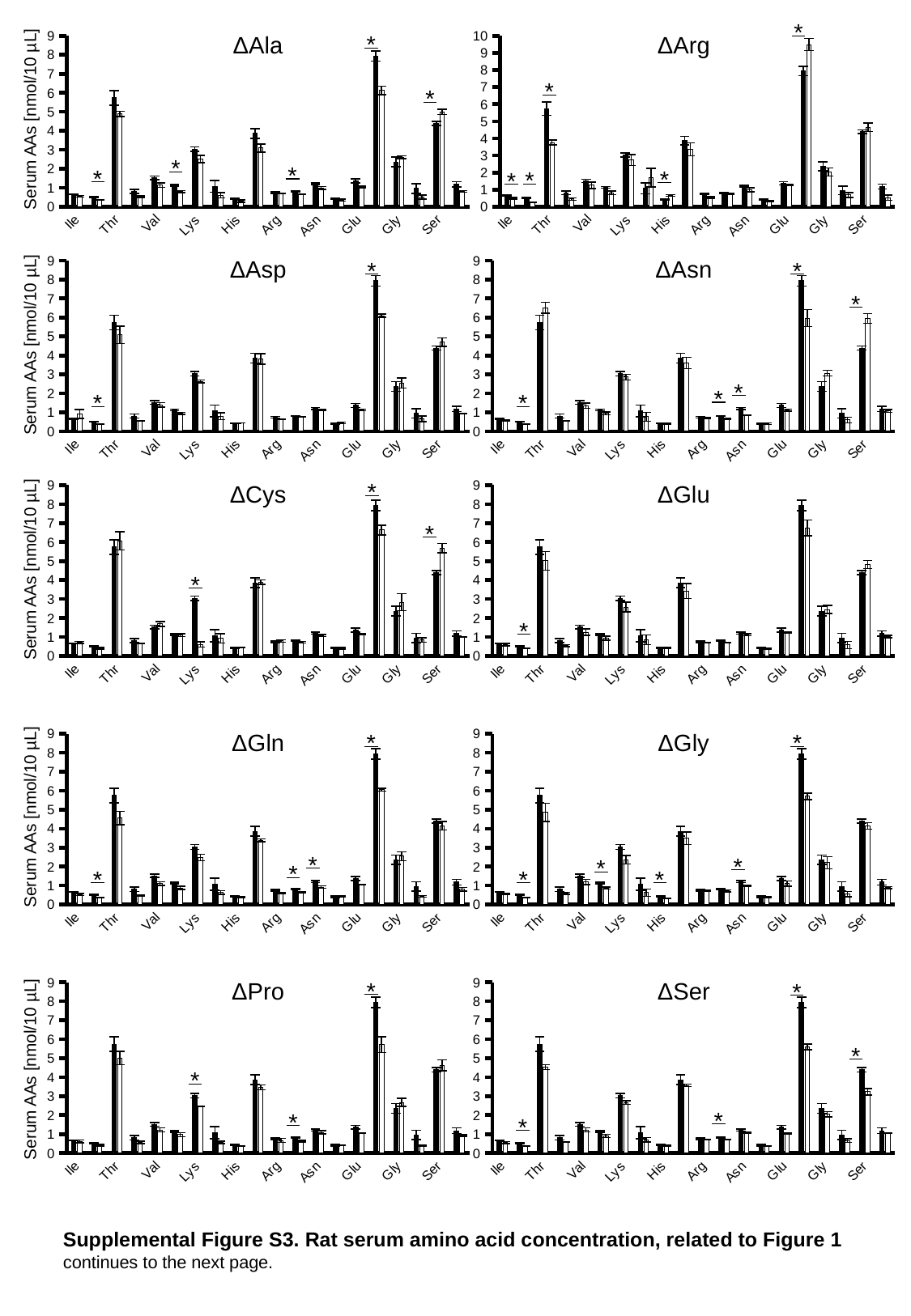

*
*
### Chart
| Category | | |
|---|---|---|
| Ile | 0.6629947811983596 | 0.5497701787641938 |
| Phe | 0.5127424672643781 | 0.35519500349979305 |
| Thr | 5.740336254712299 | 4.8934018222747 |
| Trp | 0.8058057282365031 | 0.5245227592546212 |
| Val | 1.516330666114073 | 1.1316848097363559 |
| Leu | 1.1274763637164218 | 0.801871915893697 |
| Lys | 3.030054349987205 | 2.5095080864097015 |
| Met | 1.0717299640812552 | 0.6032130630059368 |
| His | 0.42909038293339835 | 0.29223462087350055 |
| Ala | 3.86070659483901 | 3.0945945295109123 |
| Arg | 0.7457233480199355 | 0.6842599422664751 |
| Asp | 0.8085383792348607 | 0.6658336103030872 |
| Asn | 1.208845890511536 | 0.9821708546997151 |
| Cys | 0.40998024346180045 | 0.3541400549199203 |
| Glu | 1.3633755770996656 | 1.033064854326258 |
| Gln | 7.9374491586874 | 6.119282585654723 |
| Gly | 2.358812546598679 | 2.6060024622578193 |
| Pro | 0.9548825123662348 | 0.5160470246516498 |
| Ser | 4.389129553042212 | 5.000101057699784 |
| Tyr | 1.1781332174282957 | 0.8061990660488608 |ΔAla
### Chart
| Category | | |
|---|---|---|
| Ile | 0.6629947811983596 | 0.48045321781917144 |
| Phe | 0.5127424672643781 | 0.2521626110896064 |
| Thr | 5.740336254712299 | 3.7587463567371384 |
| Trp | 0.8058057282365031 | 0.44828217535530285 |
| Val | 1.516330666114073 | 1.2503631596717266 |
| Leu | 1.1274763637164218 | 0.8118165877101036 |
| Lys | 3.030054349987205 | 2.7317295087990665 |
| Met | 1.0717299640812552 | 1.6910682688991157 |
| His | 0.42909038293339835 | 0.6581305436741153 |
| Ala | 3.86070659483901 | 3.3660640307164247 |
| Arg | 0.7457233480199355 | 0.5367552890897298 |
| Asp | 0.8085383792348607 | 0.7493063539199752 |
| Asn | 1.208845890511536 | 0.9857432293700782 |
| Cys | 0.40998024346180045 | 0.33795910841386023 |
| Glu | 1.3633755770996656 | 1.2734266338434483 |
| Gln | 7.9374491586874 | 9.484509928233905 |
| Gly | 2.358812546598679 | 2.0267388901314862 |
| Pro | 0.9548825123662348 | 0.6854308798303079 |
| Ser | 4.389129553042212 | 4.645262739118686 |
| Tyr | 1.1781332174282957 | 0.5249162109306714 |ΔArg
*
*
Serum AAs [nmol/10 µL]
*
*
*
*
*
*
### Chart
| Category | | |
|---|---|---|
| Ile | 0.6629947811983596 | 0.9149474014972281 |
| Phe | 0.5127424672643781 | 0.365762992796997 |
| Thr | 5.740336254712299 | 5.08460583625484 |
| Trp | 0.8058057282365031 | 0.5542479494224505 |
| Val | 1.516330666114073 | 1.4002781228204313 |
| Leu | 1.1274763637164218 | 0.9521264004537477 |
| Lys | 3.030054349987205 | 2.627919917424557 |
| Met | 1.0717299640812552 | 0.7957920990901083 |
| His | 0.42909038293339835 | 0.4450649965685956 |
| Ala | 3.86070659483901 | 3.8164498797495057 |
| Arg | 0.7457233480199355 | 0.6233061324412547 |
| Asp | 0.8085383792348607 | 0.7588747860999286 |
| Asn | 1.208845890511536 | 1.1332512385360387 |
| Cys | 0.40998024346180045 | 0.44566482672490787 |
| Glu | 1.3633755770996656 | 1.1225053649958274 |
| Gln | 7.9374491586874 | 6.095473962236781 |
| Gly | 2.358812546598679 | 2.540473629929186 |
| Pro | 0.9548825123662348 | 0.6633405590336504 |
| Ser | 4.389129553042212 | 4.693214576547198 |
| Tyr | 1.1781332174282957 | 0.9508771524369912 |ΔAsp
### Chart
| Category | | |
|---|---|---|
| Ile | 0.6629947811983596 | 0.5872247272014833 |
| Phe | 0.5127424672643781 | 0.3675527983885534 |
| Thr | 5.740336254712299 | 6.508813082473459 |
| Trp | 0.8058057282365031 | 0.5489049705208089 |
| Val | 1.516330666114073 | 1.347995904025203 |
| Leu | 1.1274763637164218 | 0.9464244946584198 |
| Lys | 3.030054349987205 | 2.8588340471274827 |
| Met | 1.0717299640812552 | 0.7721590852542048 |
| His | 0.42909038293339835 | 0.4018855967051779 |
| Ala | 3.86070659483901 | 3.599734463550423 |
| Arg | 0.7457233480199355 | 0.6998090932775275 |
| Asp | 0.8085383792348607 | 0.656931450424473 |
| Asn | 1.208845890511536 | 0.8557069256366637 |
| Cys | 0.40998024346180045 | 0.4022634175380855 |
| Glu | 1.3633755770996656 | 1.122158609396159 |
| Gln | 7.9374491586874 | 5.967972878562265 |
| Gly | 2.358812546598679 | 3.064902349764859 |
| Pro | 0.9548825123662348 | 0.6017358009962508 |
| Ser | 4.389129553042212 | 5.9424303591052166 |
| Tyr | 1.1781332174282957 | 1.0753801160784222 |ΔAsn
*
*
*
Serum AAs [nmol/10 µL]
*
*
*
*
*
### Chart
| Category | | |
|---|---|---|
| Ile | 0.6629947811983596 | 0.7083154079798648 |
| Phe | 0.5127424672643781 | 0.40474791719921516 |
| Thr | 5.740336254712299 | 6.066752112950659 |
| Trp | 0.8058057282365031 | 0.6679315679236096 |
| Val | 1.516330666114073 | 1.6855439143812054 |
| Leu | 1.1274763637164218 | 1.093561157717867 |
| Lys | 3.030054349987205 | 0.6087336124049907 |
| Met | 1.0717299640812552 | 0.9310817896050284 |
| His | 0.42909038293339835 | 0.44542785306053795 |
| Ala | 3.86070659483901 | 3.892571227974919 |
| Arg | 0.7457233480199355 | 0.7906579520298694 |
| Asp | 0.8085383792348607 | 0.725726740272894 |
| Asn | 1.208845890511536 | 1.0659735128389547 |
| Cys | 0.40998024346180045 | 0.40353536605009666 |
| Glu | 1.3633755770996656 | 1.159949643117555 |
| Gln | 7.9374491586874 | 6.631361930093501 |
| Gly | 2.358812546598679 | 2.8318759790861283 |
| Pro | 0.9548825123662348 | 0.8518717805550561 |
| Ser | 4.389129553042212 | 5.684421264127831 |
| Tyr | 1.1781332174282957 | 1.0091641945144656 |ΔCys
### Chart
| Category | | |
|---|---|---|
| Ile | 0.6629947811983596 | 0.5979997374711064 |
| Phe | 0.5127424672643781 | 0.39990657306399235 |
| Thr | 5.740336254712299 | 5.017494243055425 |
| Trp | 0.8058057282365031 | 0.53394575918769 |
| Val | 1.516330666114073 | 1.2515978457656083 |
| Leu | 1.1274763637164218 | 0.9406500424010216 |
| Lys | 3.030054349987205 | 2.5714697373941715 |
| Met | 1.0717299640812552 | 0.8534832373479855 |
| His | 0.42909038293339835 | 0.4388175136379837 |
| Ala | 3.86070659483901 | 3.421147907699565 |
| Arg | 0.7457233480199355 | 0.7038277418321006 |
| Asp | 0.8085383792348607 | 0.7156032313906051 |
| Asn | 1.208845890511536 | 1.1258411256995928 |
| Cys | 0.40998024346180045 | 0.36786357721311697 |
| Glu | 1.3633755770996656 | 1.2177930688392038 |
| Gln | 7.9374491586874 | 6.7452356106398135 |
| Gly | 2.358812546598679 | 2.4493730182996405 |
| Pro | 0.9548825123662348 | 0.5818223810580146 |
| Ser | 4.389129553042212 | 4.824708780364916 |
| Tyr | 1.1781332174282957 | 1.0265460448417396 |ΔGlu
*
Serum AAs [nmol/10 µL]
*
*
### Chart
| Category | | |
|---|---|---|
| Ile | 0.6629947811983596 | 0.521147471893251 |
| Phe | 0.5127424672643781 | 0.3636616047503652 |
| Thr | 5.740336254712299 | 4.5568770735688355 |
| Trp | 0.8058057282365031 | 0.48436721903114366 |
| Val | 1.516330666114073 | 1.111367523652457 |
| Leu | 1.1274763637164218 | 0.877989921844221 |
| Lys | 3.030054349987205 | 2.48020517354278 |
| Met | 1.0717299640812552 | 0.6253712002923275 |
| His | 0.42909038293339835 | 0.3760484906878605 |
| Ala | 3.86070659483901 | 3.377302661211066 |
| Arg | 0.7457233480199355 | 0.6036243340108699 |
| Asp | 0.8085383792348607 | 0.6514483119123294 |
| Asn | 1.208845890511536 | 0.9212826185700127 |
| Cys | 0.40998024346180045 | 0.42623607713732464 |
| Glu | 1.3633755770996656 | 1.048389028700435 |
| Gln | 7.9374491586874 | 6.052534773752872 |
| Gly | 2.358812546598679 | 2.5471489915462504 |
| Pro | 0.9548825123662348 | 0.429343705730245 |
| Ser | 4.389129553042212 | 4.1616522068667825 |
| Tyr | 1.1781332174282957 | 0.8064259997452335 |ΔGln
### Chart
| Category | | |
|---|---|---|
| Ile | 0.6629947811983596 | 0.5520060680884026 |
| Phe | 0.5127424672643781 | 0.3665840983380317 |
| Thr | 5.740336254712299 | 4.848671338032025 |
| Trp | 0.8058057282365031 | 0.5917948992053571 |
| Val | 1.516330666114073 | 1.1837083428808017 |
| Leu | 1.1274763637164218 | 0.8664396981307112 |
| Lys | 3.030054349987205 | 2.360223108600985 |
| Met | 1.0717299640812552 | 0.6238426256056597 |
| His | 0.42909038293339835 | 0.3328271786388311 |
| Ala | 3.86070659483901 | 3.485945253422072 |
| Arg | 0.7457233480199355 | 0.7266557726894994 |
| Asp | 0.8085383792348607 | 0.7204630104324808 |
| Asn | 1.208845890511536 | 0.9696355645449715 |
| Cys | 0.40998024346180045 | 0.3872439365964286 |
| Glu | 1.3633755770996656 | 1.09674701681128 |
| Gln | 7.9374491586874 | 5.701876467891469 |
| Gly | 2.358812546598679 | 2.2010461944388933 |
| Pro | 0.9548825123662348 | 0.5445264436581627 |
| Ser | 4.389129553042212 | 4.140522050624756 |
| Tyr | 1.1781332174282957 | 0.8941990929793783 |ΔGly
*
*
Serum AAs [nmol/10 µL]
*
*
*
*
*
*
*
*
### Chart
| Category | | |
|---|---|---|
| Ile | 0.6629947811983596 | 0.6157283450832212 |
| Phe | 0.5127424672643781 | 0.4120547695707842 |
| Thr | 5.740336254712299 | 5.009438182163319 |
| Trp | 0.8058057282365031 | 0.5627555787501419 |
| Val | 1.516330666114073 | 1.2200646515937341 |
| Leu | 1.1274763637164218 | 0.9712103029761742 |
| Lys | 3.030054349987205 | 2.4634692616786524 |
| Met | 1.0717299640812552 | 0.5550663797658318 |
| His | 0.42909038293339835 | 0.376220390607406 |
| Ala | 3.86070659483901 | 3.4622261186427026 |
| Arg | 0.7457233480199355 | 0.6633747635037482 |
| Asp | 0.8085383792348607 | 0.625648685008819 |
| Asn | 1.208845890511536 | 1.0948095376534852 |
| Cys | 0.40998024346180045 | 0.42094185343067486 |
| Glu | 1.3633755770996656 | 1.0689515882609053 |
| Gln | 7.9374491586874 | 5.703690320903282 |
| Gly | 2.358812546598679 | 2.6662274976456666 |
| Pro | 0.9548825123662348 | 0.39248363569339423 |
| Ser | 4.389129553042212 | 4.6296450349474805 |
| Tyr | 1.1781332174282957 | 0.9171905787128968 |ΔPro
### Chart
| Category | | |
|---|---|---|
| Ile | 0.6629947811983596 | 0.5600234505750562 |
| Phe | 0.5127424672643781 | 0.3863029651040529 |
| Thr | 5.740336254712299 | 4.523283819731303 |
| Trp | 0.8058057282365031 | 0.5714717487293314 |
| Val | 1.516330666114073 | 1.2222720236227824 |
| Leu | 1.1274763637164218 | 0.9072837818231118 |
| Lys | 3.030054349987205 | 2.6536271497425883 |
| Met | 1.0717299640812552 | 0.7005704300430754 |
| His | 0.42909038293339835 | 0.3986708033496263 |
| Ala | 3.86070659483901 | 3.5621292988173416 |
| Arg | 0.7457233480199355 | 0.7099900670209225 |
| Asp | 0.8085383792348607 | 0.6981306551029683 |
| Asn | 1.208845890511536 | 1.083288496094468 |
| Cys | 0.40998024346180045 | 0.3676378632227106 |
| Glu | 1.3633755770996656 | 1.0174859199310957 |
| Gln | 7.9374491586874 | 5.590838381533023 |
| Gly | 2.358812546598679 | 2.044259252491967 |
| Pro | 0.9548825123662348 | 0.6548657564846977 |
| Ser | 4.389129553042212 | 3.2308477549207057 |
| Tyr | 1.1781332174282957 | 1.057210340338918 |ΔSer
*
*
Serum AAs [nmol/10 µL]
*
*
*
*
Supplemental Figure S3. Rat serum amino acid concentration, related to Figure 1
continues to the next page.

## Slide 5
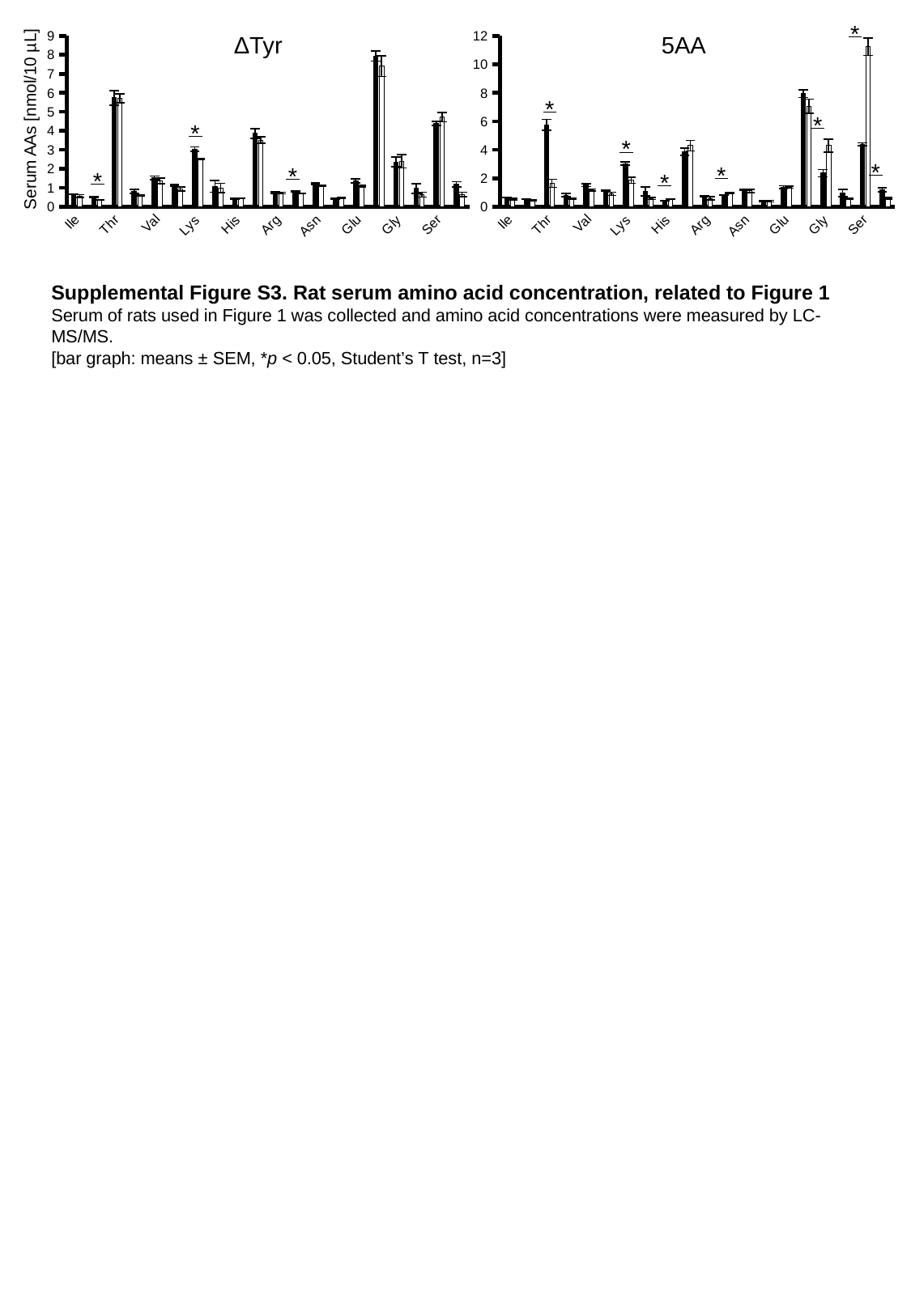

*
### Chart
| Category | | |
|---|---|---|
| Ile | 0.6629947811983596 | 0.5671234135736573 |
| Phe | 0.5127424672643781 | 0.369714032694723 |
| Thr | 5.740336254712299 | 5.713983996519542 |
| Trp | 0.8058057282365031 | 0.5984314155471786 |
| Val | 1.516330666114073 | 1.3507740373101729 |
| Leu | 1.1274763637164218 | 0.9358936669608982 |
| Lys | 3.030054349987205 | 2.496401802172985 |
| Met | 1.0717299640812552 | 0.9844557360216203 |
| His | 0.42909038293339835 | 0.4283647630221205 |
| Ala | 3.86070659483901 | 3.5081407140477814 |
| Arg | 0.7457233480199355 | 0.7133059671594412 |
| Asp | 0.8085383792348607 | 0.7005114776480864 |
| Asn | 1.208845890511536 | 1.0929287261732974 |
| Cys | 0.40998024346180045 | 0.45156239060730413 |
| Glu | 1.3633755770996656 | 1.0744408942602448 |
| Gln | 7.9374491586874 | 7.407184070193558 |
| Gly | 2.358812546598679 | 2.4021451008577674 |
| Pro | 0.9548825123662348 | 0.6393128194243548 |
| Ser | 4.389129553042212 | 4.707933854406035 |
| Tyr | 1.1781332174282957 | 0.6518372237678975 |ΔTyr
### Chart
| Category | | |
|---|---|---|
| Ile | 0.6629947811983596 | 0.5383610671059557 |
| Phe | 0.5127424672643781 | 0.4359666419206341 |
| Thr | 5.740336254712299 | 1.6429310570833708 |
| Trp | 0.8058057282365031 | 0.5668419371549956 |
| Val | 1.516330666114073 | 1.1709168792279154 |
| Leu | 1.1274763637164218 | 0.9013402203544963 |
| Lys | 3.030054349987205 | 1.8647118460693746 |
| Met | 1.0717299640812552 | 0.5756724938617593 |
| His | 0.42909038293339835 | 0.5341601893320426 |
| Ala | 3.86070659483901 | 4.289130668776824 |
| Arg | 0.7457233480199355 | 0.5766214164638201 |
| Asp | 0.8085383792348607 | 0.9771278824003908 |
| Asn | 1.208845890511536 | 1.108505252294075 |
| Cys | 0.40998024346180045 | 0.3979649670661365 |
| Glu | 1.3633755770996656 | 1.3716253743092706 |
| Gln | 7.9374491586874 | 7.059221144697176 |
| Gly | 2.358812546598679 | 4.286978672742593 |
| Pro | 0.9548825123662348 | 0.5846844644744672 |
| Ser | 4.389129553042212 | 11.233445396249541 |
| Tyr | 1.1781332174282957 | 0.5775638581089061 |5AA
*
Serum AAs [nmol/10 µL]
*
*
*
*
*
*
*
*
Supplemental Figure S3. Rat serum amino acid concentration, related to Figure 1
Serum of rats used in Figure 1 was collected and amino acid concentrations were measured by LC-MS/MS.
[bar graph: means ± SEM, *p < 0.05, Student’s T test, n=3]

## Slide 6
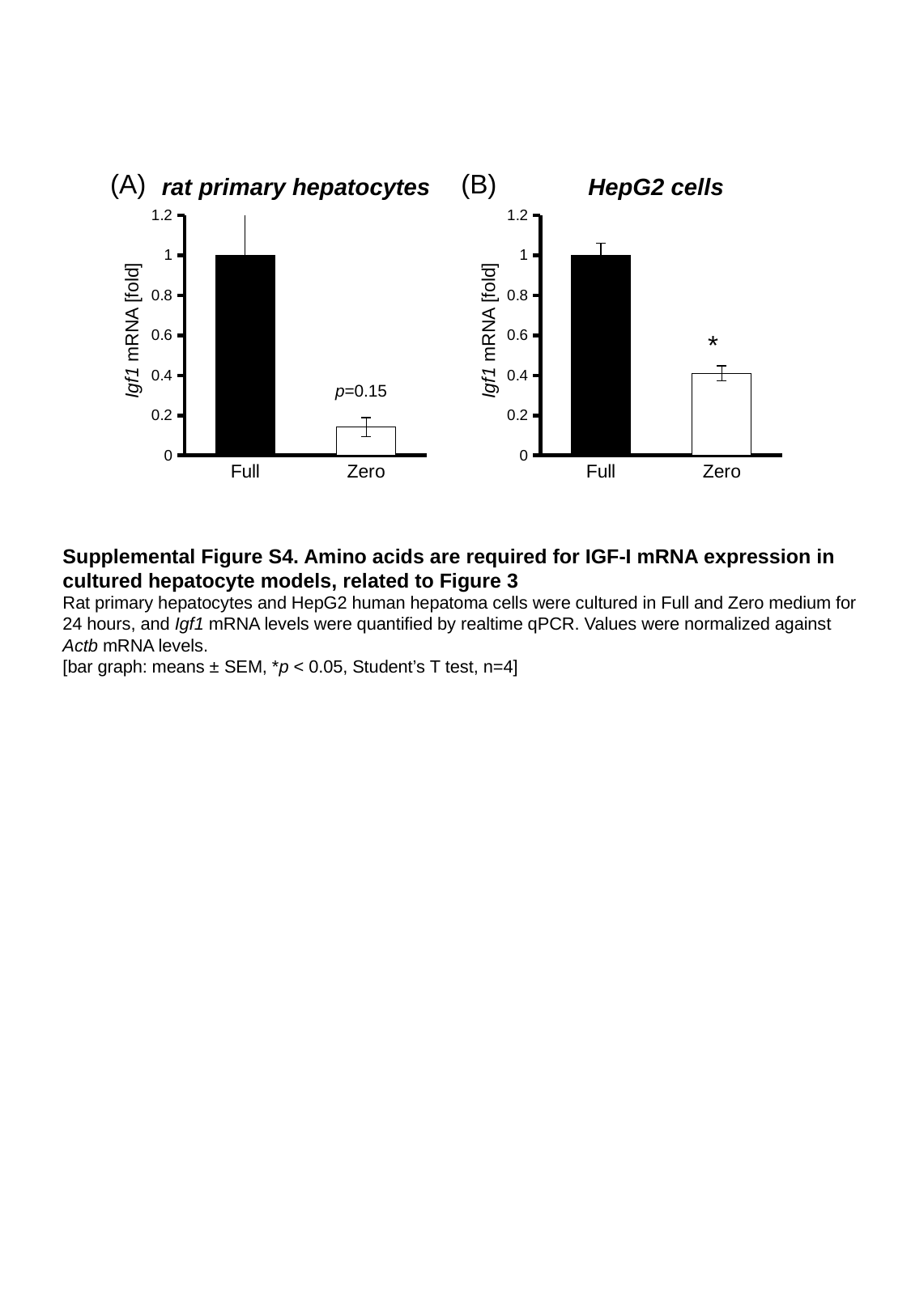

(A)
(B)
rat primary hepatocytes
HepG2 cells
### Chart
| Category | |
|---|---|
| Full | 1.0 |
| Zero | 0.14225107300116288 |
### Chart
| Category | |
|---|---|
| Full | 1.0 |
| Zero | 0.4107421245101564 |Igf1 mRNA [fold]
Igf1 mRNA [fold]
*
p=0.15
Supplemental Figure S4. Amino acids are required for IGF-I mRNA expression in cultured hepatocyte models, related to Figure 3
Rat primary hepatocytes and HepG2 human hepatoma cells were cultured in Full and Zero medium for 24 hours, and Igf1 mRNA levels were quantified by realtime qPCR. Values were normalized against Actb mRNA levels.
[bar graph: means ± SEM, *p < 0.05, Student’s T test, n=4]

## Slide 7
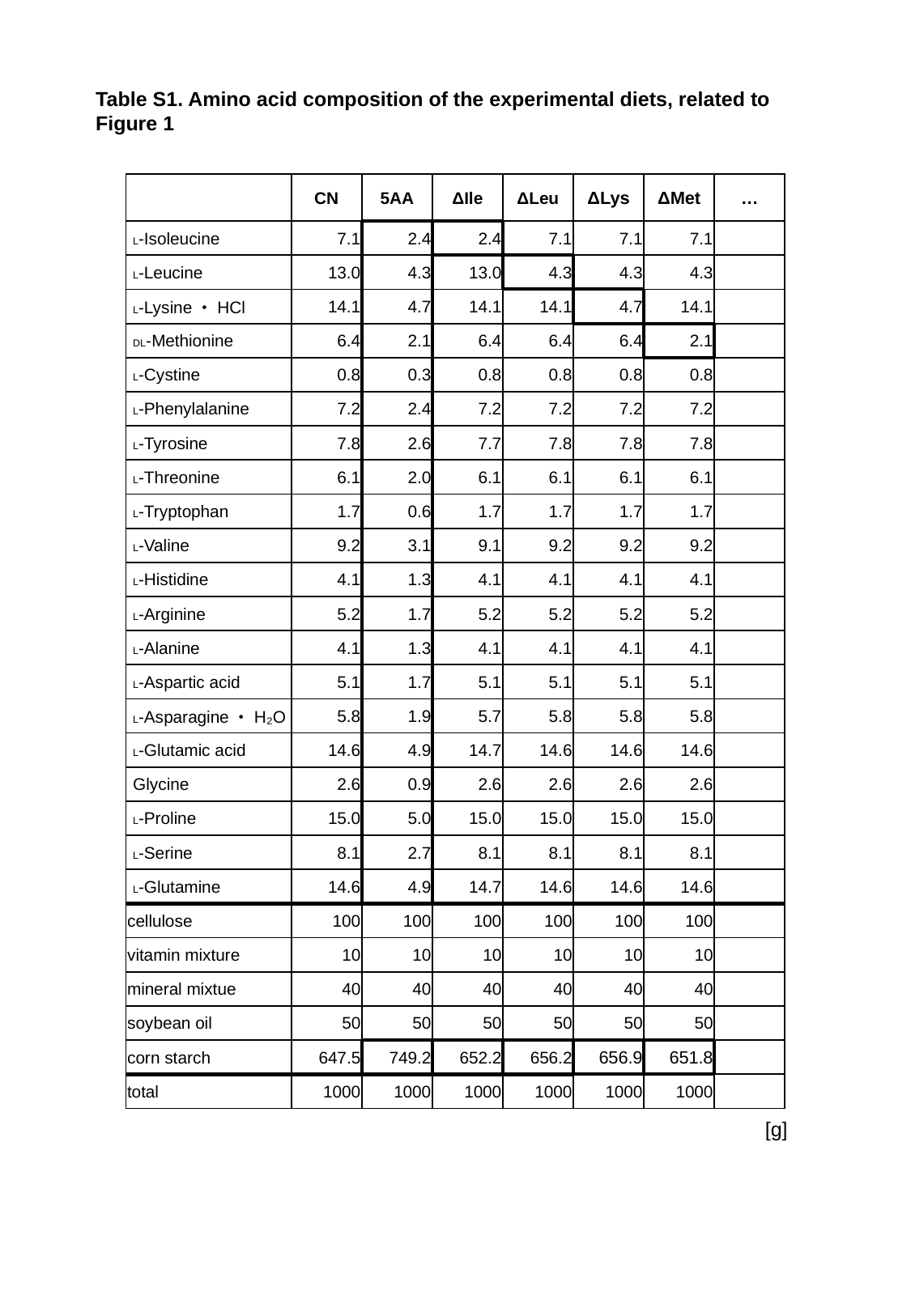

Table S1. Amino acid composition of the experimental diets, related to Figure 1
| | CN | 5AA | ΔIle | ΔLeu | ΔLys | ΔMet | … |
| --- | --- | --- | --- | --- | --- | --- | --- |
| L-Isoleucine | 7.1 | 2.4 | 2.4 | 7.1 | 7.1 | 7.1 | |
| L-Leucine | 13.0 | 4.3 | 13.0 | 4.3 | 4.3 | 4.3 | |
| L-Lysine・HCl | 14.1 | 4.7 | 14.1 | 14.1 | 4.7 | 14.1 | |
| DL-Methionine | 6.4 | 2.1 | 6.4 | 6.4 | 6.4 | 2.1 | |
| L-Cystine | 0.8 | 0.3 | 0.8 | 0.8 | 0.8 | 0.8 | |
| L-Phenylalanine | 7.2 | 2.4 | 7.2 | 7.2 | 7.2 | 7.2 | |
| L-Tyrosine | 7.8 | 2.6 | 7.7 | 7.8 | 7.8 | 7.8 | |
| L-Threonine | 6.1 | 2.0 | 6.1 | 6.1 | 6.1 | 6.1 | |
| L-Tryptophan | 1.7 | 0.6 | 1.7 | 1.7 | 1.7 | 1.7 | |
| L-Valine | 9.2 | 3.1 | 9.1 | 9.2 | 9.2 | 9.2 | |
| L-Histidine | 4.1 | 1.3 | 4.1 | 4.1 | 4.1 | 4.1 | |
| L-Arginine | 5.2 | 1.7 | 5.2 | 5.2 | 5.2 | 5.2 | |
| L-Alanine | 4.1 | 1.3 | 4.1 | 4.1 | 4.1 | 4.1 | |
| L-Aspartic acid | 5.1 | 1.7 | 5.1 | 5.1 | 5.1 | 5.1 | |
| L-Asparagine・H₂O | 5.8 | 1.9 | 5.7 | 5.8 | 5.8 | 5.8 | |
| L-Glutamic acid | 14.6 | 4.9 | 14.7 | 14.6 | 14.6 | 14.6 | |
| Glycine | 2.6 | 0.9 | 2.6 | 2.6 | 2.6 | 2.6 | |
| L-Proline | 15.0 | 5.0 | 15.0 | 15.0 | 15.0 | 15.0 | |
| L-Serine | 8.1 | 2.7 | 8.1 | 8.1 | 8.1 | 8.1 | |
| L-Glutamine | 14.6 | 4.9 | 14.7 | 14.6 | 14.6 | 14.6 | |
| cellulose | 100 | 100 | 100 | 100 | 100 | 100 | |
| vitamin mixture | 10 | 10 | 10 | 10 | 10 | 10 | |
| mineral mixtue | 40 | 40 | 40 | 40 | 40 | 40 | |
| soybean oil | 50 | 50 | 50 | 50 | 50 | 50 | |
| corn starch | 647.5 | 749.2 | 652.2 | 656.2 | 656.9 | 651.8 | |
| total | 1000 | 1000 | 1000 | 1000 | 1000 | 1000 | |
[g]

## Slide 8
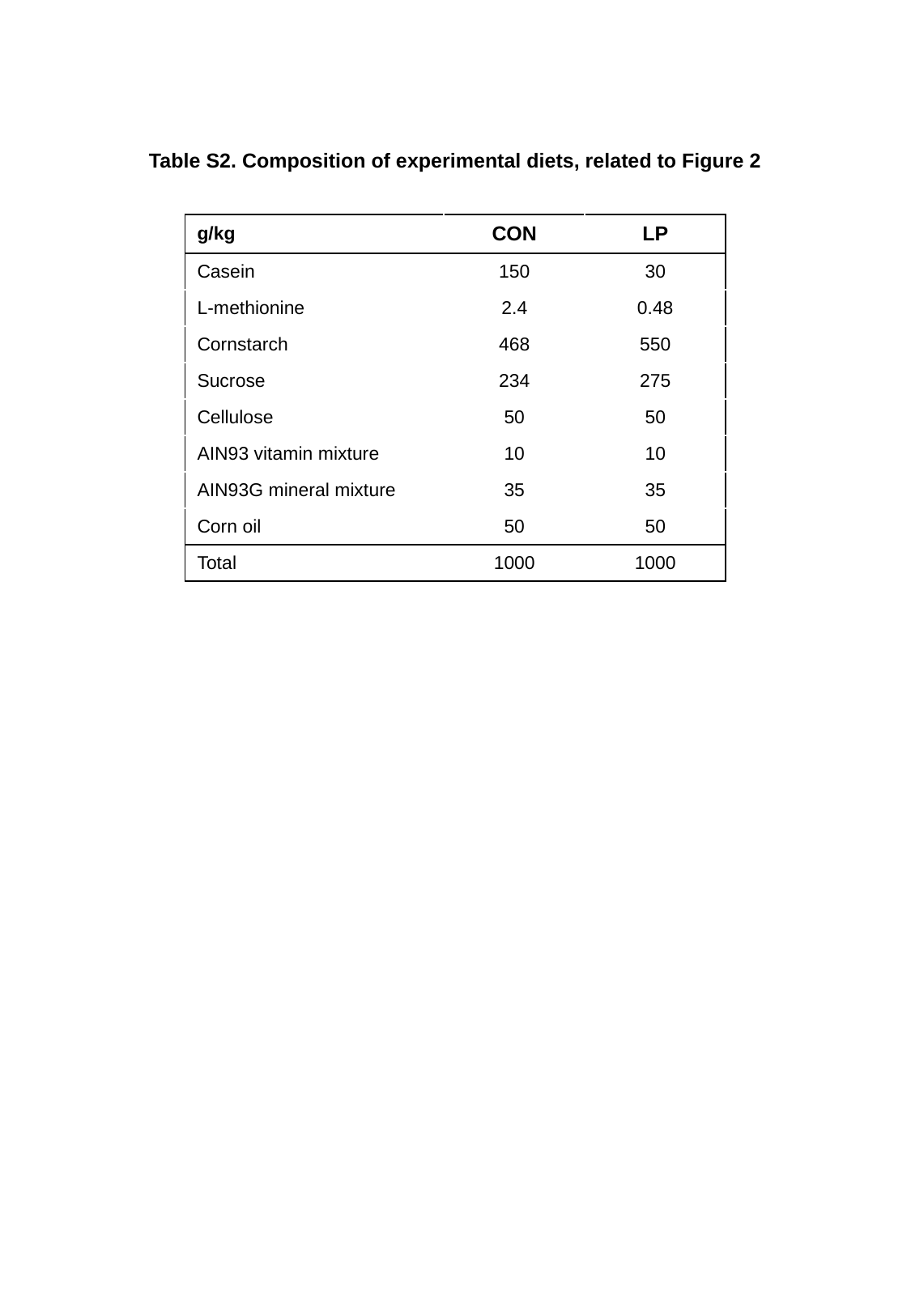

Table S2. Composition of experimental diets, related to Figure 2
| g/kg | CON | LP |
| --- | --- | --- |
| Casein | 150 | 30 |
| L-methionine | 2.4 | 0.48 |
| Cornstarch | 468 | 550 |
| Sucrose | 234 | 275 |
| Cellulose | 50 | 50 |
| AIN93 vitamin mixture | 10 | 10 |
| AIN93G mineral mixture | 35 | 35 |
| Corn oil | 50 | 50 |
| Total | 1000 | 1000 |

## Slide 9
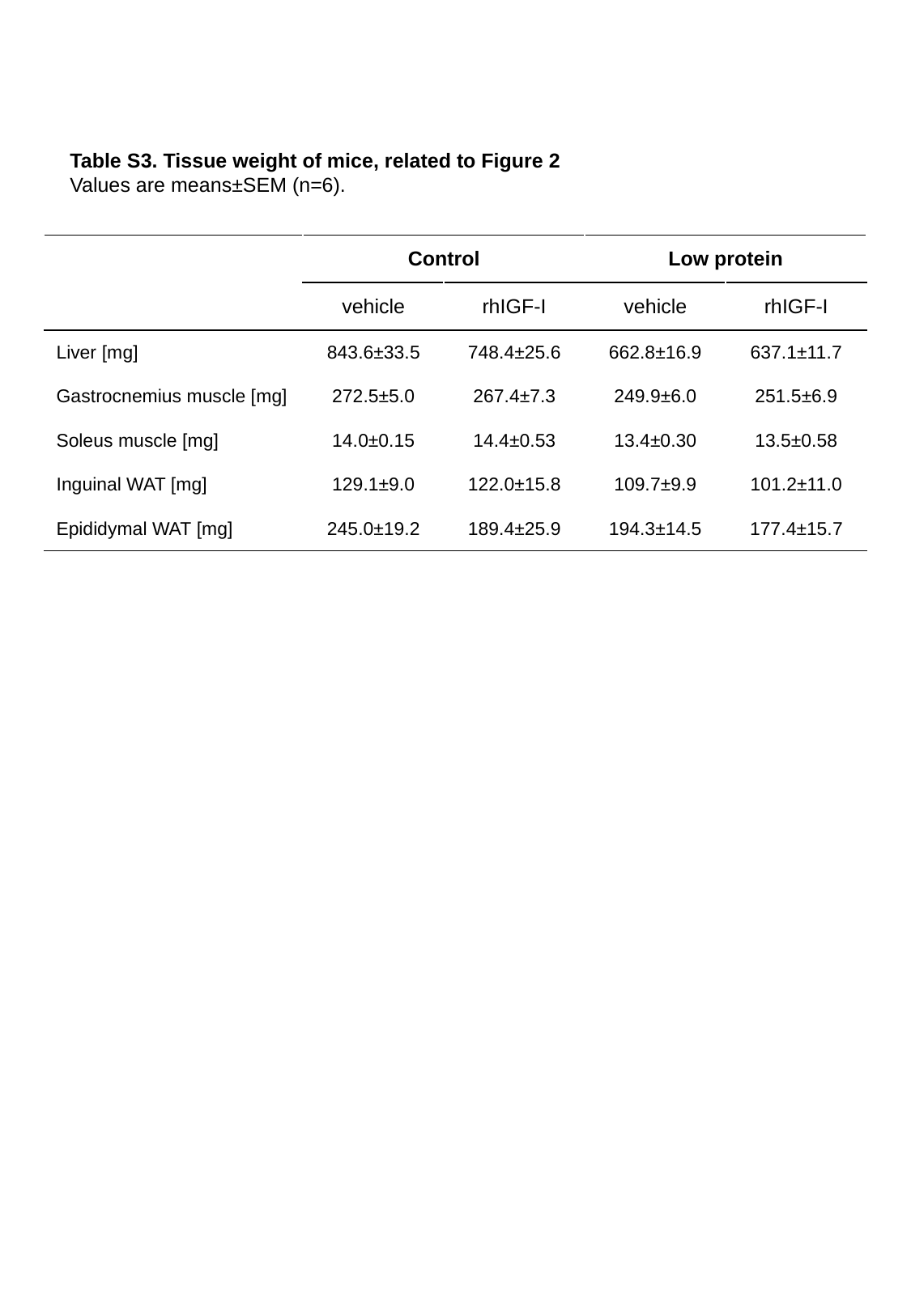

Table S3. Tissue weight of mice, related to Figure 2
Values are means±SEM (n=6).
| | Control | | Low protein | |
| --- | --- | --- | --- | --- |
| | vehicle | rhIGF-I | vehicle | rhIGF-I |
| Liver [mg] | 843.6±33.5 | 748.4±25.6 | 662.8±16.9 | 637.1±11.7 |
| Gastrocnemius muscle [mg] | 272.5±5.0 | 267.4±7.3 | 249.9±6.0 | 251.5±6.9 |
| Soleus muscle [mg] | 14.0±0.15 | 14.4±0.53 | 13.4±0.30 | 13.5±0.58 |
| Inguinal WAT [mg] | 129.1±9.0 | 122.0±15.8 | 109.7±9.9 | 101.2±11.0 |
| Epididymal WAT [mg] | 245.0±19.2 | 189.4±25.9 | 194.3±14.5 | 177.4±15.7 |

## Slide 10
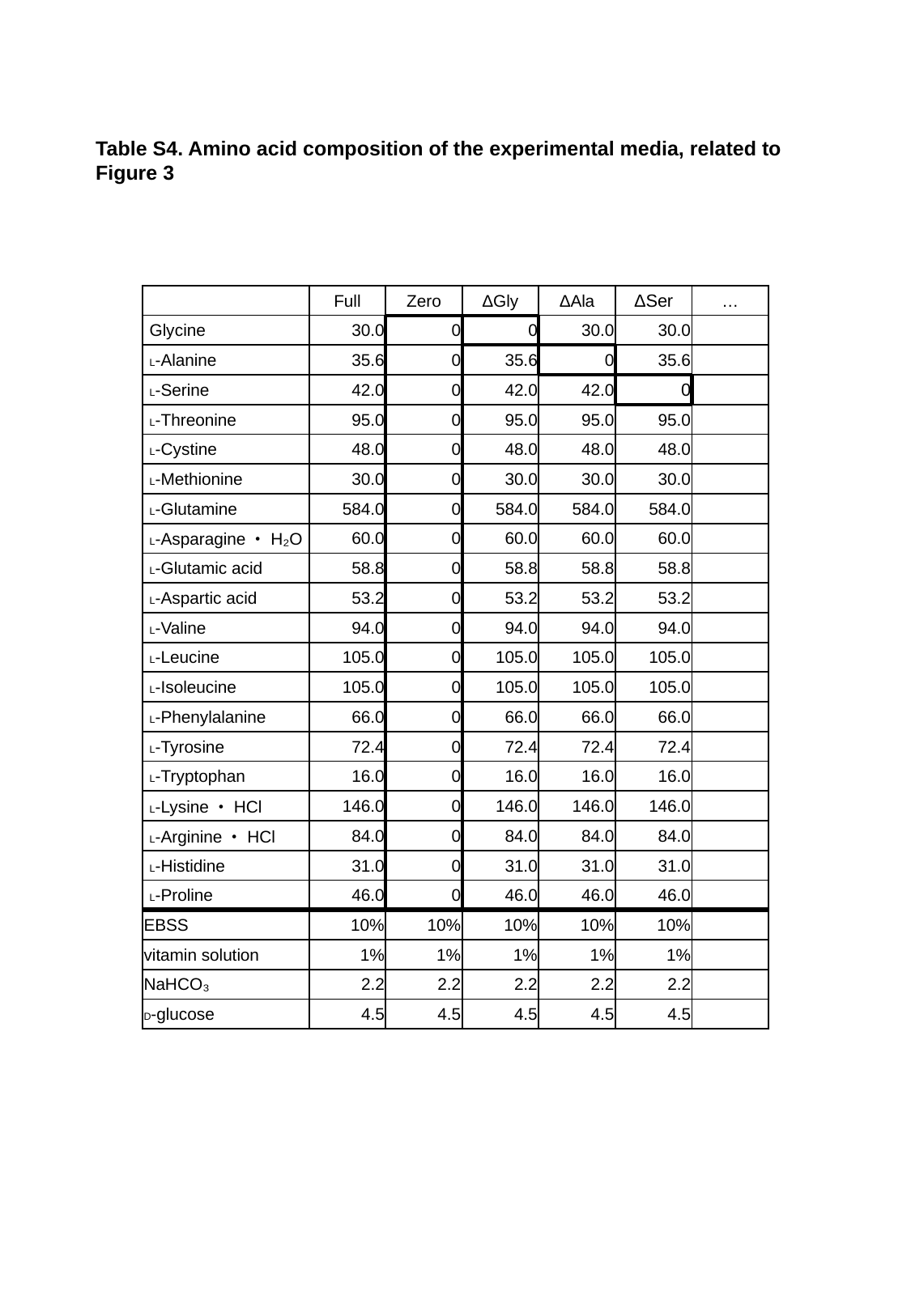

Table S4. Amino acid composition of the experimental media, related to Figure 3
| | Full | Zero | ΔGly | ΔAla | ΔSer | … |
| --- | --- | --- | --- | --- | --- | --- |
| Glycine | 30.0 | 0 | 0 | 30.0 | 30.0 | |
| L-Alanine | 35.6 | 0 | 35.6 | 0 | 35.6 | |
| L-Serine | 42.0 | 0 | 42.0 | 42.0 | 0 | |
| L-Threonine | 95.0 | 0 | 95.0 | 95.0 | 95.0 | |
| L-Cystine | 48.0 | 0 | 48.0 | 48.0 | 48.0 | |
| L-Methionine | 30.0 | 0 | 30.0 | 30.0 | 30.0 | |
| L-Glutamine | 584.0 | 0 | 584.0 | 584.0 | 584.0 | |
| L-Asparagine・H₂O | 60.0 | 0 | 60.0 | 60.0 | 60.0 | |
| L-Glutamic acid | 58.8 | 0 | 58.8 | 58.8 | 58.8 | |
| L-Aspartic acid | 53.2 | 0 | 53.2 | 53.2 | 53.2 | |
| L-Valine | 94.0 | 0 | 94.0 | 94.0 | 94.0 | |
| L-Leucine | 105.0 | 0 | 105.0 | 105.0 | 105.0 | |
| L-Isoleucine | 105.0 | 0 | 105.0 | 105.0 | 105.0 | |
| L-Phenylalanine | 66.0 | 0 | 66.0 | 66.0 | 66.0 | |
| L-Tyrosine | 72.4 | 0 | 72.4 | 72.4 | 72.4 | |
| L-Tryptophan | 16.0 | 0 | 16.0 | 16.0 | 16.0 | |
| L-Lysine・HCl | 146.0 | 0 | 146.0 | 146.0 | 146.0 | |
| L-Arginine・HCl | 84.0 | 0 | 84.0 | 84.0 | 84.0 | |
| L-Histidine | 31.0 | 0 | 31.0 | 31.0 | 31.0 | |
| L-Proline | 46.0 | 0 | 46.0 | 46.0 | 46.0 | |
| EBSS | 10% | 10% | 10% | 10% | 10% | |
| vitamin solution | 1% | 1% | 1% | 1% | 1% | |
| NaHCO₃ | 2.2 | 2.2 | 2.2 | 2.2 | 2.2 | |
| D-glucose | 4.5 | 4.5 | 4.5 | 4.5 | 4.5 | |

## Slide 11
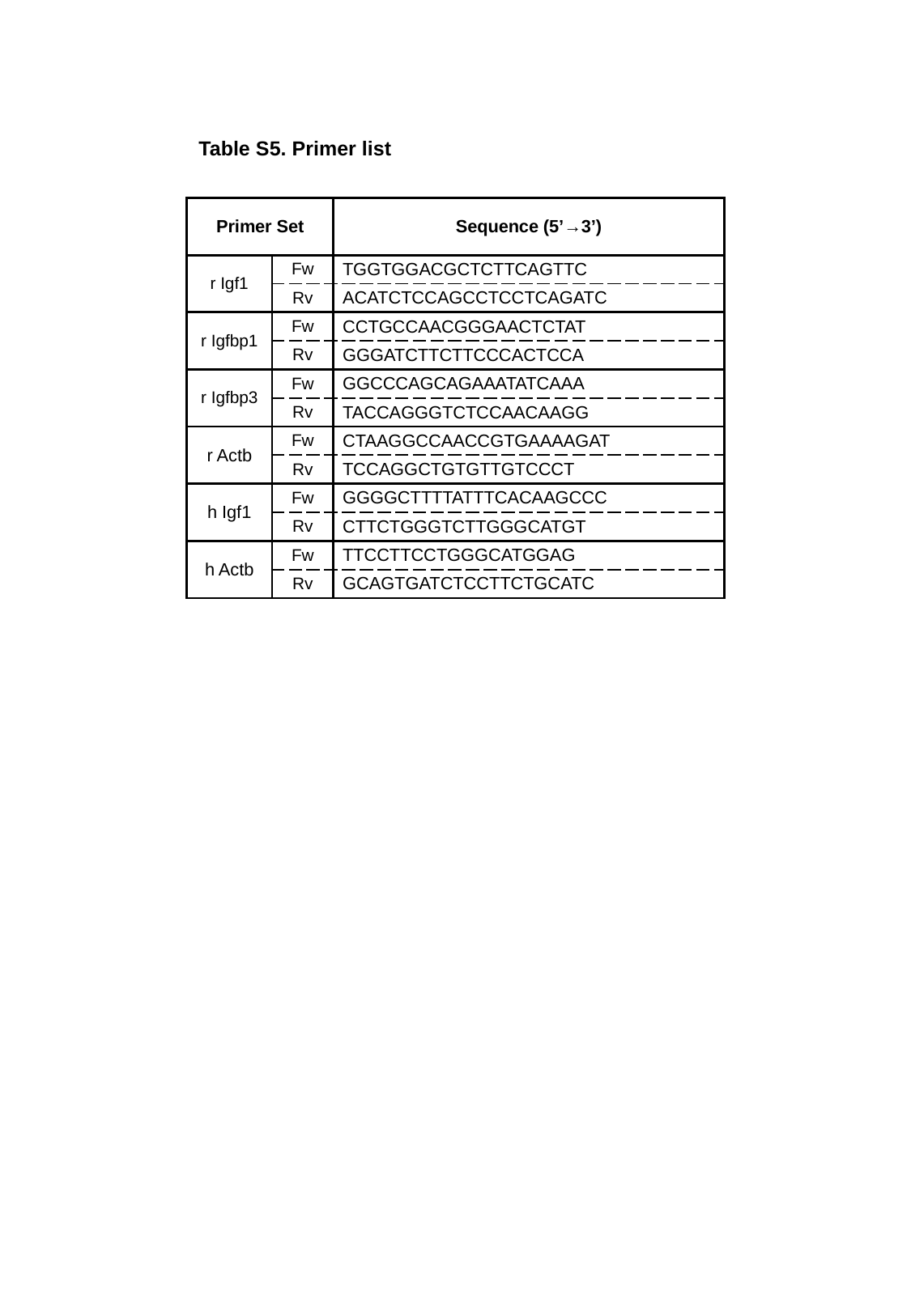

Table S5. Primer list
| Primer Set | | Sequence (5’→3’) |
| --- | --- | --- |
| r Igf1 | Fw | TGGTGGACGCTCTTCAGTTC |
| #1 Rv | Rv | ACATCTCCAGCCTCCTCAGATC |
| r Igfbp1 | Fw | CCTGCCAACGGGAACTCTAT |
| #2 Rv | Rv | GGGATCTTCTTCCCACTCCA |
| r Igfbp3 | Fw | GGCCCAGCAGAAATATCAAA |
| | Rv | TACCAGGGTCTCCAACAAGG |
| r Actb | Fw | CTAAGGCCAACCGTGAAAAGAT |
| #3 Rv | Rv | TCCAGGCTGTGTTGTCCCT |
| h Igf1 | Fw | GGGGCTTTTATTTCACAAGCCC |
| #4 Rv | Rv | CTTCTGGGTCTTGGGCATGT |
| h Actb | Fw | TTCCTTCCTGGGCATGGAG |
| #5 Rv | Rv | GCAGTGATCTCCTTCTGCATC |
